# Dual stimulation of antigen presenting cells using carbon nanotube-based vaccine delivery system for cancer immunotherapy

**DOI:** 10.1016/j.biomaterials.2016.07.005

**Published:** 2016-10

**Authors:** Hatem A.F.M. Hassan, Lesley Smyth, Julie T.-W. Wang, Pedro M. Costa, Kulachelvy Ratnasothy, Sandra S. Diebold, Giovanna Lombardi, Khuloud T. Al-Jamal

**Affiliations:** a Institute of Pharmaceutical Science, Faculty of Life Sciences & Medicine, King's College London, Franklin−Wilkins Building, London SE1 9NH, United Kingdom; b Immunoregulation Laboratory, MRC Centre for Transplantation, King's College London, Guy's Hospital, London SE1 9RT, United Kingdom; c Division of Immunology, Infection, and Inflammatory Diseases, King's College London, Guy's Hospital, London SE1 9RT, United Kingdom

**Keywords:** Carbon nanotubes, Dendritic cells, Nanomedicine, Vaccine delivery, Cancer vaccines

## Abstract

Although anti−cancer immuno−based combinatorial therapeutic approaches have shown promising results, efficient tumour eradication demands further intensification of anti−tumour immune response. With the emerging field of nanovaccinology, multi−walled carbon nanotubes (MWNTs) have manifested prominent potentials as tumour antigen nanocarriers. Nevertheless, the utilization of MWNTs in co−delivering antigen along with different types of immunoadjuvants to antigen presenting cells (APCs) has not been investigated yet. We hypothesized that harnessing MWNT for concurrent delivery of cytosine−phosphate−guanine oligodeoxynucleotide (CpG) and anti-CD40 Ig (αCD40), as immunoadjuvants, along with the model antigen ovalbumin (OVA) could potentiate immune response induced against OVA−expressing tumour cells. We initially investigated the effective method to co−deliver OVA and CpG using MWNT to the APC. Covalent conjugation of OVA and CpG prior to loading onto MWNTs markedly augmented the CpG−mediated adjuvanticity, as demonstrated by the significantly increased OVA−specific T cell responses *in vitro* and in C57BL/6 mice. αCD40 was then included as a second immunoadjuvant to further intensify the immune response. Immune response elicited *in vitro* and *in vivo* by OVA, CpG and αCD40 was significantly potentiated by their co−incorporation onto the MWNTs. Furthermore, MWNT remarkably improved the ability of co−loaded OVA, CpG and αCD40 in inhibiting the growth of OVA−expressing B16F10 melanoma cells in subcutaneous or lung pseudo−metastatic tumour models. Therefore, this study suggests that the utilization of MWNTs for the co−delivery of tumour−derived antigen, CpG and αCD40 could be a competent approach for efficient tumours eradication.

## 1 Introduction

Cancer therapeutic vaccines rely on the ability of professional antigen presenting cells (APCs), specifically dendritic cells (DCs), to detect, process and then present administered tumour−antigens *via* the major histocompatibility complex molecules class (MHC) II or I to CD4^+^ or CD8^+^ T cells, respectively, leading to anti−tumour immune responses induction [1]. However, tumour−induced immunosuppression and abundance of immunosuppressive regulatory T cells in the tumour micro−environment hinder the immune system to effectively eradicate established tumours [2]. This could be overcome by the use of combinatorial immunotherapeutic approaches, for instance by administration of tumour antigens along with different types of immunoadjuvants, rather than the single immunotherapeutic ones [3].

Carbon nanotubes (CNTs) have been developed as needle−like nanoscopic carriers capable of improving therapeutic agents delivery to the intracellular compartments *via* energy−dependent and/or passive mechanisms of cellular uptake [4], [5], [6]. We have previously demonstrated that altering the surface chemistry of multi−walled CNTs (MWNTs) conjugated to the model antigen ovalbumin (OVA) can affect the extent of their cellular internalization into APCs, and thus the intensity of the resulting immune responses elicited *in vitro* and *in vivo* [7]. As a delivery vector for tumour antigens, MWNTs have markedly improved antitumour immune response against breast or liver cancer–derived tumour proteins *in vitro* [8] or *in vivo* [9], respectively. Single−walled CNTs (SWNTs) conjugation with the immunoadjuvant cytosine−phosphate−guanine oligodeoxynucleotide (CpG) enhanced the CpG−induced stimulatory activities *in vitro* [10] and amplified the anti−tumour response in glioma−bearing mice [11], [12]. Despite the demonstrated proficiencies of CNTs as an efficient delivery vector for antigen or immunoadjuvant, the utilization of CNTs to co−deliver antigen along with various types of immunoadjuvants has not been studied yet.

Agonist for Toll−like receptors (TLRs) expressed by APCs have shown marked capabilities in augmenting the antigen−specific immune response *via* various mechanisms, including the ability to enhance antigen presentation by APCs [13]. CpG, an agonist for endosomal TLR9, has been included in various cancer vaccine formulations tested in clinical trials [14]. The co−internalization of both antigen and TLR agonist by the same APC has been shown to influence the TLR agonist−mediated improvement of antigen presentation, thus the induced T cell responses. Yarovinsky et al. showed that potent induction of antigen−specific CD4^+^ T cell response in mice required the activation of TLR11 and antigen presentation *via* MHC II to occur “in cis” in the same DC instead of separate DCs [15]. Wilson et al. demonstrated the significance of APC activation by TLR agonists at the time of antigen uptake showing that pre−treatment of mice with CpG reduces the ability of DCs to take up and present viral antigens to the CD8^+^ T cells [16]. Posing additional complexity, Blander et al. reported that more efficient antigen presentation by DCs could be achieved following the internalization of antigen and TLR4 agonist into the same rather than separate phagosome(s) *in vitro* [17]. In light of these studies, we hypothesized that designing an efficient method to co−incorporate antigen and CpG onto MWNT could improve their concomitant delivery to APC, and thus the induction of an antigen-specific immune response.

APCs express a number of receptors known as tumour necrosis factor receptors (TNFRs) such as CD40. Anti-CD40 Ig (αCD40), an agonist for the CD40 co-stimulation molecule, has exhibited potential benefits in amplifying antigen−specific immune responses [18], [19]. It has been reported that DC stimulation with αCD40 chemically conjugated to peptide antigens increased the DC capacity to induce antigen−specific CD8^+^ T cell response *in vitro* [20], [21]. This has been attributed to the demonstrated αCD40 ability to intracellularly target the conjugated antigen to the early endosomes of DCs. Antigen routing to the early endosomes of DCs has shown to facilitate antigen proteasomal degradation, loading onto MHC I and, subsequently, presentation to CD8^+^ T cells [22]. Thereby, by utilizing the αCD40−mediated enhancement of antigen presentation, stimulation of DCs with MWNT loaded with αCD40 in addition to OVA could further improve the induction of OVA−specific CD8^+^ T cell response [23]. CD40 interaction with αCD40 has been found to provide APCs with the CD4^+^ T cell−derived licensing signals required for CD8^+^ T cell stimulation. This has been demonstrated by the ability of administered αCD40 to restore antigen specific CD8^+^ T cell response in CD4^+^ T cell−depleted mice [24], [25]. In addition, for an efficient CD8^+^ T cell response induction, the process of antigen recognition by both CD4^+^ and CD8^+^ T cells has to occur *via* the same APC [26]. Hence, theoretically, higher immune response intensity could be achieved using delivery approaches that co−deliver the antigen and αCD40 signal to the same APC.

We hypothesized that CpG and αCD40 co−incorporation onto MWNT carrying the model antigen OVA would synergistically and significantly improve the OVA−specific immune responses, and effectively retard the growth of OVA−expressing B16F10 melanoma cells in solid or pseudo−metastatic tumour models.

## 2 Materials and methods

### 2.1 Mice

All the experiments involving the animal use were carried out in accordance with the project and personal license authorized by the UK Home Office and UKCCCR Guidelines (1998). The C57BL/6 mice were purchased from Harlan (UK). The OT1 Rag^−/−^ and OT2 Rag^−/−^ mice were maintained at Charles River (UK). All experiments use were performed using female 6–8 weeks old mice.

### 2.2 Synthesis of S^−/+^(OVA−CpG) or (OVA)S^−/+^(CpG)

**Scheme 1:**
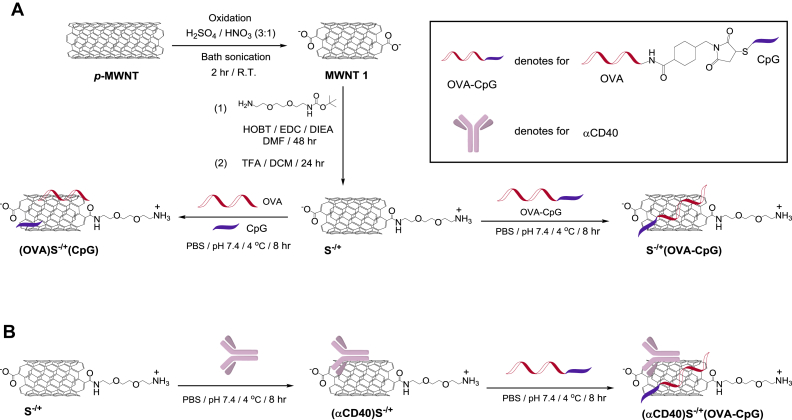
**Synthesis of the conjugates**. **(A)** Synthesis of (OVA)S^−/+^(CpG) or S^−/+^(OVA−CpG) conjugates. *p*−MWNT was oxidized using acidic mixture yielding MWNT **1**. The carboxylic acid moieties of MWNT **1** were reacted with Boc−protected amine−terminated spacer *via* amide coupling reaction yielding S^−/+^. (OVA)S^−/+^(CpG) was synthesized by the simultaneous addition of OVA and CpG to S^−/+^, while S^−/+^(OVA−CpG) was synthesized by reacting the OVA−CpG with S^−/+^. **(B)** Synthesis of (αCD40)S^−/+^(OVA−CpG). αCD40 was first conjugated with S^−/+^ yielding (αCD40)S^−/+^ that following conjugation with OVA−CpG yielded (αCD40)S^−/+^(OVA−CpG).

Synthesis of chemically functionalized MWNT has been described before and is shown in Scheme 1 [7]. Briefly, pristine MWNTs (*p*−MWNTs) (20–30 nm diameter, 0.5–2 μm length, Nanostructured and Amorphous Materials, USA) were oxidized using acidic mixture, followed by incorporation of amine−terminated spacer using amide coupling reaction yielding a functionalized MWNT named S^−/+^. The synthesis of OVA−CpG is illustrated in Scheme S1 and described in Supplementary Information [27], [28]. For the synthesis of (OVA)S^−/+^(CpG), 0.5 ml of PBS (PAA Laboratories Ltd, UK) containing 1 mg OVA (EndoGrade^®^ Ovalbumin, Hyglos GmbH, Germany) and 1.1 mg CpG (phosphorothioate ODN CpG 1668 (5′−(TCCATGACGTTCCTGATGCT)−3′), Eurogentec, Belgium) were mixed with a dispersion of 2 mg S^−/+^ in 2 ml PBS. For the synthesis of S^−/+^(OVA−CpG), OVA−CpG containing 1 mg OVA and 1.1 mg CpG in 0.5 ml PBS was mixed with a dispersion of 2 mg S^−/+^ in 2 ml PBS. Both reactions were mixed for 8 h at 4 °C. The reaction mixtures were briefly sonicated then vacuum filtered through 0.22 μm polycarbonate membrane filter (Isopore™ Membrane, Merck Millipore, Germany). The solids recovered were re−dispersed in 2.5 ml PBS and the obtained dispersion was briefly sonicated and then vacuum filtered. Unreacted OVA and CpG contained in the collected filtrates were quantified using bicinchoninic acid protein (BCA) assay reagent (Fisher Scientific, UK) and NanoDrop (ND−1000 spectrophotometer, NanoDrop Technologies, USA), respectively, as described in Supplementary Information. The recovered S^−/+^(OVA−CpG) or (OVA)S^−/+^(CpG) solids were further washed with methanol (Fisher Scientific, UK) and then vacuum filtered through 0.22 μm polycarbonate membrane filter, dried and recovered. Synthesized conjugates were characterized using thermogravimetric analysis (TGA) and polyacrylamide gel electrophoresis (PAGE) that were performed as described before [7].

### 2.3 Synthesis of (αCD40)S^−/+^(OVA−CpG)

To a dispersion of 3 mg S^−/+^ in 2 ml PBS, 1 mg of αCD40 (purified rat anti−mouse CD40 monoclonal antibody, BD Biosciences, USA) in 0.5 ml PBS was added. The reaction was mixed for 8 h at 4 °C. The reaction mixture was briefly sonicated then vacuum filtered through 0.22 μm polycarbonate membrane filter. The solids recovered were re−dispersed in 2.5 ml PBS and the obtained dispersion was briefly sonicated and then vacuum filtered. Unreacted αCD40 contained in the collected filtrates was quantified using BCA assay as described in Supplementary Information. The recovered (αCD40)S^−/+^ solids were washed with methanol, vacuum filtered through 0.22 μm polycarbonate membrane filter, dried and re−dispersed in 2 ml PBS. To the (αCD40)S^−/+^ dispersion, OVA−CpG containing 1 mg OVA and 1.1 mg CpG in 0.5 ml PBS was added. The reaction was mixed for 8 h at 4 °C. The reaction mixture was briefly sonicated then vacuum filtered through 0.22 μm polycarbonate membrane filter. The solids recovered were re−dispersed in 2.5 ml PBS and the obtained dispersion was briefly sonicated and then vacuum filtered. Unreacted OVA and CpG contained in the collected filtrates were quantified using BCA assay and NanoDrop, respectively, as described in Supplementary Information. The recovered (αCD40)S^−/+^(CpG) solids were further washed with methanol and then vacuum filtered through 0.22 μm polycarbonate membrane filter, dried and recovered.

### 2.4 Assessment of OVA presentation induced by (OVA)S^−/+^(CpG) or S^−/+^(OVA−CpG) treated BM−DCs *in vitro*

DCs were generated from the bone marrow of C57BL/6 mice and characterized for their purity as previously described [7]. Bone marrow−derived DCs (BM−DCs) were incubated for 24 h with OVA, mixture of unconjugated OVA and CpG (referred to as OVA + CpG), OVA−CpG, (OVA)S^−/+^(CpG) or S^−/+^(OVA−CpG) each containing 5 μg/ml OVA. The used doses were determined from the optimization studies described in Supplementary Information (Figure S1). BM−DCs were incubated, as a control, with S^−/+^ alone at concentrations equivalent to those contained in (OVA)S^−/+^(CpG) or S^−/+^(OVA−CpG) (20–38 μg/ml). Treated BM−DCs were harvested, washed several times with RPMI 1640 and gamma−irradiated using Cesium−137 at 3000 Gy for 10 min. CD4^+^ and CD8^+^ T cells were isolated from the OT−II and OT−I mice spleen, respectively, and characterized for their purity as described before [7]. In 96−well round−bottom plate, 25 × 10^3^ of CD4^+^ or CD8^+^ T cells were co−cultured with the BM−DCs at 1:4 ratio in a total volume of 200 μl complete medium per well. The BM−DCs: T cell co−culture ratio was determined from previous optimization studies [7]. As a control, CD4^+^ or CD8^+^ T cells were cultured alone or with naïve BM−DCs. Cultured cells were maintained for 3 days at 37 °C. For the last 18 h of incubation, 50 μl of the supernatants were removed and replaced with a fresh 50 μl of complete medium containing 1 μCi of ^3^H−thymidine (Thymidine (Methyl−^3^H), Perkin Elmer, USA). T cell proliferation was assessed by measuring the incorporated ^3^H−thymidine emitted radiation using liquid scintillation counter (Wallac 1205 Betaplate) [7]. The levels of IFN−γ in the supernatants collected from BM−DCs co−cultured with CD4^+^ or CD8^+^ T cells were quantified using anti−mouse IFN−γ sandwich ELISA kit (eBioscience, USA) following the manufacturer's protocol. The absorbance of each well was measured at 450 nm using a plate reader (FLUOstar Omega, BMG LABTECH, Germany).

### 2.5 Assessment of OVA presentation induced by (αCD40)S^−/+^(OVA−CpG) treated BM−DCs *in vitro*

BM−DCs were incubated for 24 h with mixture of unconjugated αCD40 and OVA−CpG (referred to as αCD40 + OVA−CpG), mixture of unconjugated αCD40 and S^−/+^(OVA−CpG) (referred to as αCD40 + S^−/+^(OVA−CpG)) or (αCD40)S^−/+^(OVA−CpG), each containing 0.5 μg/ml of both OVA and CpG, and 1.8 μg/ml αCD40. As a control BM−DCs were incubated for 24 h with OVA−CpG or S^−/+^(OVA−CpG), each containing 1 μg/ml OVA. CD8^+^ T cell proliferation and IFN−γ production were then determined using ^3^H−thymidine incorporation assay and ELISA as described before, respectively.

### 2.6 Assessment of the immune response induced by (OVA)S^−/+^(CpG), S^−/+^(OVA−CpG) or (αCD40)S^−/+^(OVA−CpG) in mice using *in vivo* CTL assay

C57BL/6 mice (n = 3–5) were immunized, *via* the footpad injection, with OVA−CpG, (OVA)S^−/+^(CpG) or S^−/+^(OVA−CpG) each containing 6 μg OVA in 50 μl PBS. Alternatively, mice were injected *via* the footpad with αCD40 + OVA−CpG or (αCD40)S^−/+^(OVA−CpG), each containing 3 μg of both OVA and CpG, and 10 μg αCD40 in 50 μl PBS. Mice injected with PBS were used as a control. The *in vivo* cytotoxic T lymphocyte (CTL) assay was performed following previously described method [7]. Briefly, a 1:1 splenocytes mixture consisting of 0.5 μM CFSE (carboxyfluorescein diacetate succinimidyl ester, eBioscience, USA) −labelled and SIINFEKL−pulsed splenocytes (referred to as 0.5 μM CFSE^SIINFEKL^) and 5 μM CFSE−labelled un−pulsed splenocytes (referred to as 5 μM CFSE^no SIINFEKL^), were administered in immunized mice or mice injected with PBS *via* the tail vein at 10 × 10^6^ cells per 200 μl per mouse, on the 8th day post immunization. At 18 h post−injection, mice were scarified; spleens were harvested and digested in collagenase/DNase solution. The percentage of SIINFEKL−pulsed and un−pulsed splenocytes, induced by each treatment, in the harvested splenocytes was determined using flow cytometry. Antigen−specific killing was calculated using the following equation:

$$\left[1-\frac{\left[\text{Percentage}\;\text{of}\;0.5\;\text{μM}\;{\text{CFSE}}^{\text{SIINFEKL}}\right]}{\left[\text{Percentage}\;\text{of}\;5\;\text{μM}\;{\text{CFSE}}^{\text{no}\;\text{SIINFEKL}}\right]}\right]\times 100$$

### 2.7 Assessment of the anti−tumour response induced by (αCD40)S^−/+^(OVA−CpG) in subcutaneous tumour model

Luciferase−transfected melanoma B16F10 cells were obtained from Perkin Elmer (USA) and were transduced with vesicular stomatitis virus G pseudotyped retrovirus encoding green fluorescence protein (GFP)−tagged OVA [27], [29]. GFP positive cells were then sorted as single cells using GFP filter. C57BL/6 mice were subcutaneously inoculated in both flanks with 2.5 × 10^5^ OVA−expressing and luciferase−transfected B16F10 (OVA−B16F10−Luc) cells. On the 7th day post tumour inoculation, mice were randomly assigned to 5 groups (n = 7). On the 7th and 14th days post tumour inoculation, mice were immunized *via* footpad injection with S^−/+^(OVA−CpG), αCD40 + OVA−CpG, αCD40 + S^−/+^(OVA−CpG) or (αCD40)S^−/+^(OVA−CpG), each containing 6 μg OVA, 6 μg CpG and/or 21 μg αCD40 in 50 μl PBS. PBS injected mice were used as untreated controls. A calliper was used to measure the tumour length (L) and width (W), and the tumour volume was calculated using the following equation: Tumour volume = 0.52 × W^2^ × L. Mice were sacrificed when the tumour volume reached 1000 mm^3^.

### 2.8 Assessment of anti−tumour response induced by (αCD40)S^−/+^(OVA−CpG) in lung pseudo−metastatic tumour model

C57BL/6 mice were intravenously inoculated with 2.5 × 10^5^ OVA−B16F10−Luc cells. On the 4th day post tumour inoculation, mice were randomly assigned to 3 groups (n = 6–8). On the 4th and 9th days post tumour inoculation, mice were immunized *via* footpad injection with S^−/+^(OVA−CpG) or (αCD40)S^−/+^(OVA−CpG) containing 6 μg OVA, 6 μg CpG and/or 21 μg αCD40. PBS injected mice were used as untreated controls. Tumour growth was monitored by detecting the bioluminescence emitted from the inoculated OVA−B16F10−Luc cells following D−Luciferin (Perkin Elmer, USA) injection. Every 3–4 days post tumour inoculation, mice were anesthetized and subcutaneously injected with D−Luciferin (150 mg/kg) in PBS. Imaging was performed using IVIS Lumina III and images analysis was conducted with Living Image^®^ 4.3.1 Service Pack 2 software (Perkin Elmer, USA).

### 2.9 Histological analysis

Heart, lung, kidney, spleen and lymph nodes were isolated from mice at sacrifice. Isolated tissues were fixed using 10% neutral buffer formalin (Sigma, UK) and tissue sections were stained using haematoxylin and eosin (H & E) or Neutral Red (NR) following the standard staining protocols of the Royal Veterinary College (UK). Images of the stained histological specimens were captured using Leica DM 1000 LED Microscope (Leica Microsystems, UK) connected to CDD digital camera (Qimaging, UK).

### 2.10 Statistical analysis

Results are expressed as mean value ± standard deviation (S.D.), unless otherwise stated. Statistical analysis was performed using GraphPad Prism version 5.01 (USA). Statistical differences were determined using one−way ANOVA followed by Bonferroni post−test.

## 3 Results

### 3.1 Synthesis and characterization of (OVA)S^−/+^(CpG), S^−/+^(OVA−CpG) and (αCD40)S^−/+^(OVA−CpG) conjugates

As depicted in Scheme 1A, the length of *p*−MWNTs was shortened by oxidation reaction using sulphuric and nitric acids mixture and bath sonication, yielding MWNT **1**. This step was followed by partial neutralization of the incorporated negatively charged carboxylic acid moieties using an amine terminated spacer, yielding S^−/+^. We have previously reported that this functionalization approach, compared to other chemical functionalization methods, yields a functionalized MWNT (S^−/+^) capable of significantly improving the loaded antigen internalization by APCs *in vitro* and *in vivo* [7].

Utilizing the ability of CNTs to non−covalently interact with proteins [30] and ssDNA [31], OVA and CpG were incorporated onto S^−/+^ surface using two distinct methods (Scheme 1A). (OVA)S^−/+^(CpG) was synthesized by mixing an aqueous dispersion of S^−/+^ with OVA and CpG. Alternatively, OVA and CpG were covalently conjugated to yield OVA−CpG (Scheme S1) prior to the reaction with S^−/+^, yielding S^−/+^(OVA−CpG). The third conjugate, (αCD40)S^−/+^(OVA−CpG), was prepared by prior mixing of αCD40 with S^−/+^ followed by OVA−CpG loading (Scheme 1B).

**Fig. 1:**
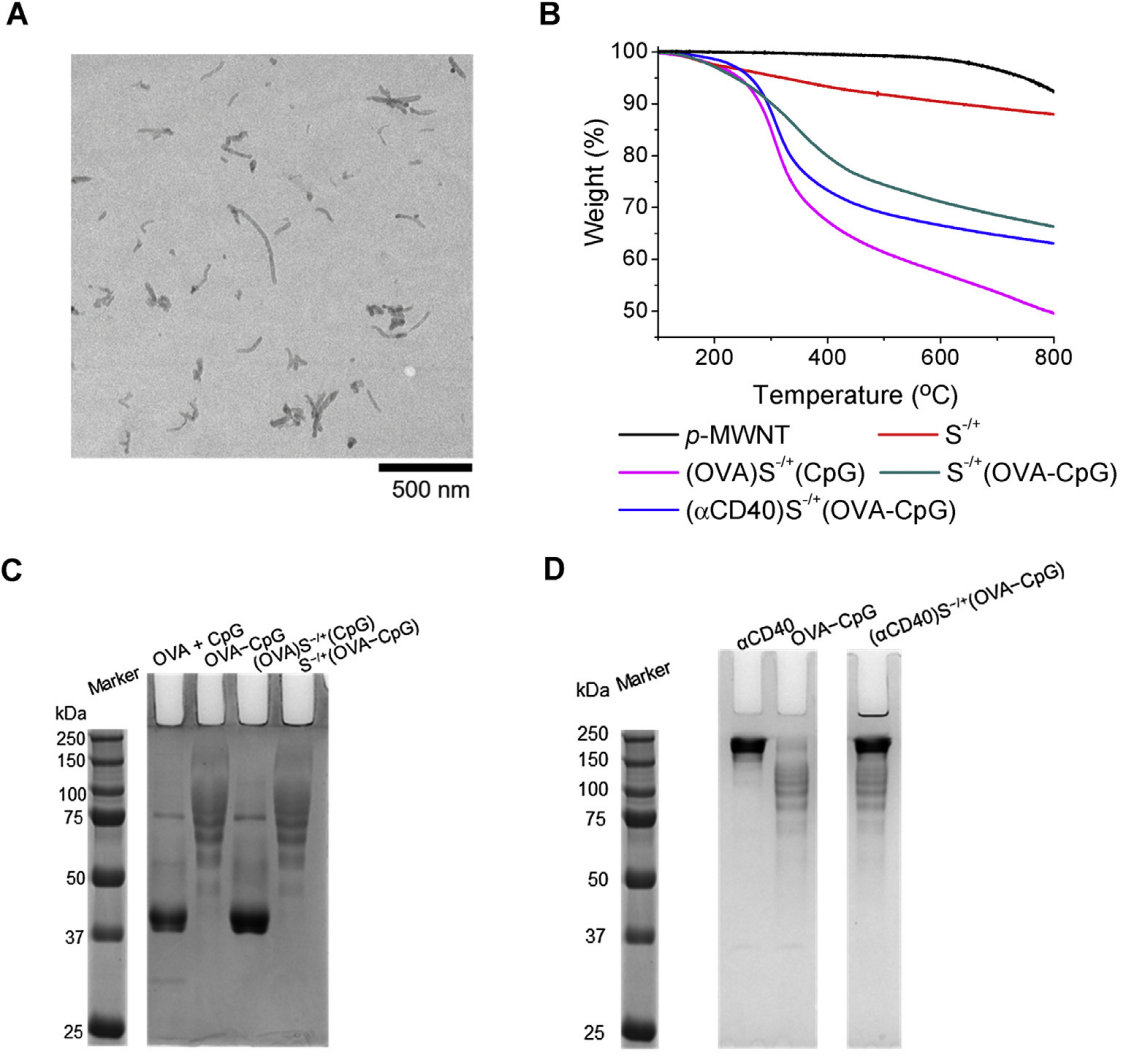
**Characterization of the conjugates**. **(A)** Representative TEM image of an aqueous dispersion of S^−/+^. **(B)** Thermogravimetric profiles. S^−/+^ or the conjugates, of know weights, were subjected to increasing temperatures and the weight loss was measured at the increased temperature. **(C)** PAGE of (OVA)S^−/+^(CpG) or S^−/+^(OVA−CpG). Free OVA, or OVA contained in the conjugates, each at 10 μg OVA, were loaded in the appropriate lane of 10% native, non−reducing gel. **(D)** PAGE of (αCD40)S^−/+^(OVA−CpG). OVA−CpG conjugate containing 3 μg OVA, 10 μg of αCD40 or (αCD40)S^−/+^(OVA−CpG) containing 3 μg OVA and 10 μg αCD40 were loaded in the appropriate lane of 10% native, non−reducing gel. Bands were detected by gel staining with Coomassie Brilliant blue.

The zeta potential values of S^−/+^, (OVA)S^−/+^(CpG) and S^−/+^(OVA−CpG) were found to be −7.27, −43.7 and −41.9 mV, respectively (Table S1). Transmission electron microscopy (TEM) revealed individualized nanotubes with a mean length of 122 ± 82 nm (Fig. 1A). OVA or αCD40 loading was quantified using a BCA assay [7], while CpG quantification was performed using NanoDrop Spectrophotometer [11]. The loading values and loading efficiency are summarized in Table 1 and Table S2, respectively. OVA: CpG molar ratios of 1:10, 1:7.3 and 1:7.4 were reported for (OVA)S^−/+^(CpG), S^−/+^(OVA−CpG) and (αCD40)S^−/+^(OVA−CpG), respectively. Stability studies were carried out up to 7 days by stirring in PBS at 37 C^◦^; the stability of the loaded cargo was confirmed (Figure S2).

OVA, CpG and/or αCD40 loading onto S^−/+^ was also confirmed using TGA [32]. TGA was performed under inert gas (nitrogen) by exposing the tested samples to gradually increasing temperature (up to 800 °C). The graphitic structure of the pristine CNT (*p*-MWNT) is stable against sublimation within the applied temperatures. However, surface defects and impurities such as amorphous carbon (that constitute approximately 2% of *p*-MWNT) are less stable and thermally decompose by sublimation at 600 °C [33], [34]. Organic functional groups decomposition also occurs at temperatures lower than 600 °C. It was previously reported that thermally degraded functional groups or biomolecules decompose mainly into carbon dioxide, carbon monoxide, ammonia [35].

The density of the loaded functional groups and biomolecules is directly related to the sample weight loss 600 °C, as a result of thermal decomposition. As demonstrated in Fig. 1B, a greater reduction in the thermal stability was observed for S^−/+^ compared to *p*−MWNT as a result of decomposition of the functional groups. Expectedly, (OVA)S^−/+^(CpG), (αCD40)S^−/+^(OVA−CpG) and S^−/+^(OVA−CpG) achieved higher weight losses than S^−/+^, in the same order. This observation could be assigned to the fact that (OVA)S^−/+^(CpG) possessed the highest content of incorporated biomolecules followed by (αCD40)S^−/+^(OVA−CpG) then S^−/+^(OVA−CpG) (Table 1). TGA confirmed the success of chemical modification of S^−/+^ and loading of OVA, CpG and αCD40 onto S^−/+^.

PAGE electrophoresis was employed to visualize the loaded OVA and/or αCD40. Similar to unconjugated OVA, the OVA contained in (OVA)S^−/+^(CpG) appeared as an intense band of ∼45 kDa (Fig. 1C). In case of OVA−CpG or S^−/+^(OVA−CpG), an increase in OVA molecular weight was observed (>45 kDa) due to successful conjugation with CpG. Exposing (αCD40)S^−/+^(OVA−CpG) to gel electrophoresis confirmed the presence of αCD40 as a main intense band of ∼150 kDa and CpG−conjugated OVA bands (Fig. 1D).

### 3.2 Loading of OVA−CpG conjugate onto S^−/+^ offers more potent *in vitro* antigen presentation than the loading of unconjugated OVA and CpG

**Fig. 2:**
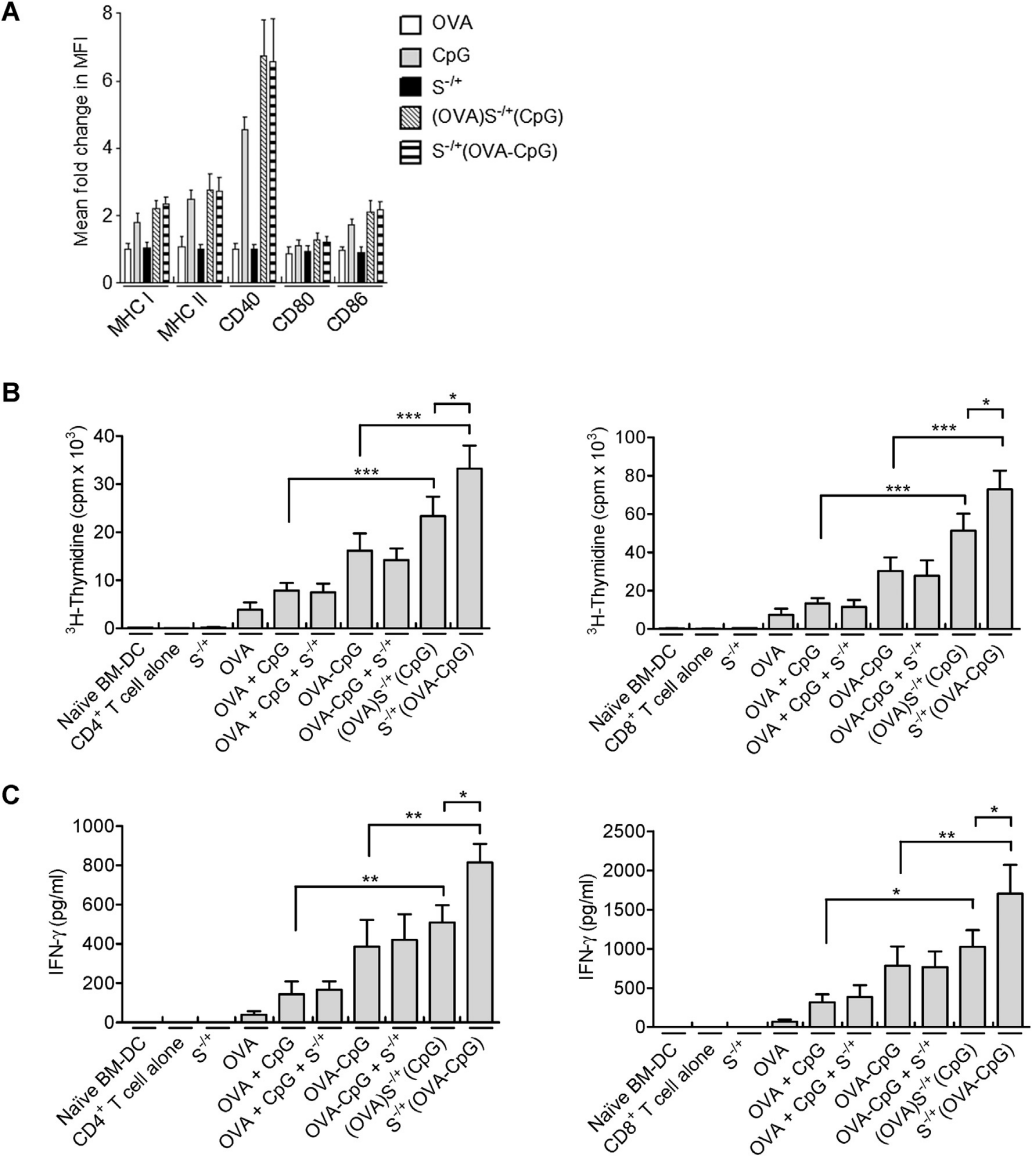
**Assessment of BM−DC maturation and OVA presentation induced by treatment with (OVA)S**^**−/+**^**(CpG) or S**^**−/+**^**(OVA−CpG) *in vitro***. **(A)** Effect of (OVA)S^−/+^(CpG) or S^−/+^(OVA−CpG) on BM−DC maturation. BM−DCs were incubated for 24 h with 5 μg/ml CpG, OVA, (OVA)S^−/+^(CpG) or S^−/+^(OVA−CpG), each contained 5 μg/ml OVA. BM−DCs were stained with fluorescently labelled specific antibodies against MHC I, MHC II, CD40, CD80 or CD86, and cell analysis was performed using flow cytometry. The mean fluorescence intensity (MFI) of the positive CD11c−expressing BM−DCs was measured to assess the fold change in the expression of each marker with respect to the naïve BM−DCs, results represent the mean ± S.D. **(B, C)** OVA presentation by (OVA)S^−/+^(CpG) or S^−/+^(OVA−CpG) treated BM−DCs. BM−DCs were incubated for 24 h with OVA + CpG, OVA−CpG, (OVA)S^−/+^(CpG) or S^−/+^(OVA−CpG), each contained 5 μg/ml OVA. Treated BM−DCs were co−cultured with CD4^+^ or CD8^+^ T cells isolated from the spleen of OT−2 or OT−1 C57BL/6 mice, respectively, at 1:4 ratio for 3 days. On the last 18 h of incubation, CD4^+^ T cells (**B**, left) or CD8^+^ T cells (**B**, right) were pulsed with 1 μCi of ^3^H−thymidine and the proliferation was measured using ^3^H−thymidine incorporation assay. The content of IFN−γ in the supernatants of the proliferating CD4^+^ T cells (**C**, left) or CD8^+^ T cells (**C**, right) was quantified using ELISA. Measurements were performed in triplicates for each condition, results represent the mean ± S.D. *P < 0.05, **P < 0.01, ***P < 0.001.

To determine the effect of (OVA)S^−/+^(CpG) or S^−/+^(OVA−CpG) on the maturation of DC, the synthesized conjugates were incubated with BM−DCs for 24 h and the expression of MHC as well as co-stimulatory molecules were determined using specific antibodies and flow cytometry as described in Supplementary Information. Similar to the CpG treatment alone, incubation of BM−DCs with (OVA)S^−/+^(CpG) or S^−/+^(OVA−CpG) increased the expression of MHC I, MHC II, CD40 and CD86 (Fig. 2A and Figure S3) with no significant differences seen between groups. OVA−treated BM−DCs showed no signs of maturation. Incubation of BM−DCs with S^−/+^ alone has been shown previously to not affect the expression of these molecules [7]. Taken together the data suggest that maturation of BM−DCs induced by (OVA)S^−/+^(CpG) or S^−/+^(OVA−CpG) was CpG−dependant.

Next, the efficiency of the two approaches in enhancing OVA presentation by BM−DCs was assessed *in vitro* using OVA−specific transgenic CD4^+^ or CD8^+^ T cells. After incubation of BM−DCs with the S^−/+^ based conjugates (containing 5 μg/ml OVA), (OVA)S^−/+^(CpG) or S^−/+^(OVA−CpG) significantly enhanced OVA−specific T cells proliferation as compared to their control treatments, namely OVA + CpG or OVA−CpG (p < 0.001), respectively (Fig. 2B). However, treatment with S^−/+^(OVA−CpG) resulted in elevated responses compared to (OVA)S^−/+^(CpG) (p < 0.05). This was further confirmed with IFN−γ cytokine production profiles (Fig. 2C). Similar T cell responses were obtained when BM−DCs were treated with the synthesized conjugates containing a lower dose of 2.5 μg/ml OVA (data not shown).

These observations indicated that loading OVA and CpG onto S^−/+^ in the form of a conjugate can lead to enhanced OVA presentation by BM−DCs *in vitro*, compared to loading unconjugated OVA and CpG.

### 3.3 Immunization with S^−/+^ loaded with OVA−CpG elicits potent cellular and humoral immune responses

The capability of (OVA)S^−/+^(CpG) or S^−/+^(OVA−CpG) to induce a cell−mediated immune response *in vivo* was determined using an *in vivo* CTL assay [27]. In these experiments, the OVA immunization dose used in (OVA)S^−/+^(CpG), S^−/+^(OVA−CpG) or their controls was 6 μg, given our observation that in C57BL/6 mice treated with various doses of OVA−CpG, a measureable OVA−specific CTL immune response was detected at OVA content of 6 μg (Figure S4).

**Fig. 3:**
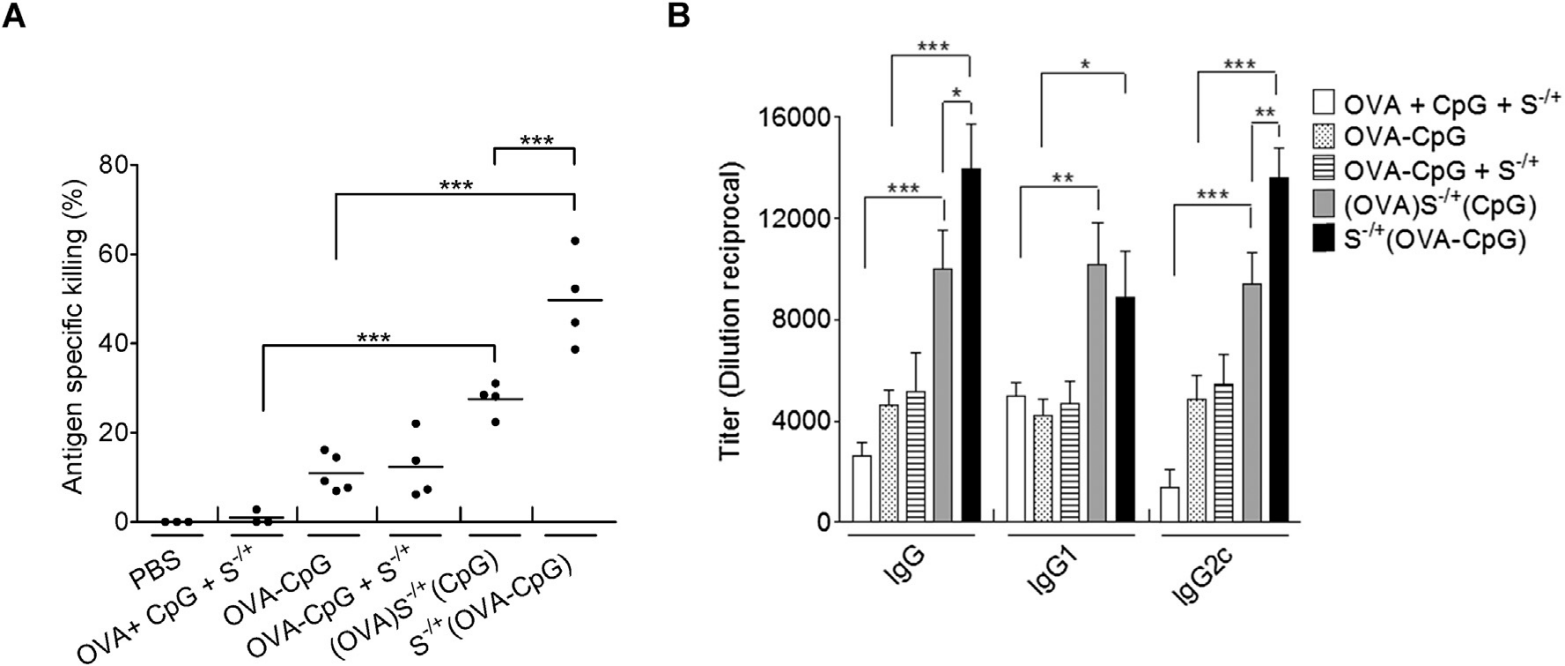
**Assessment of immune response induced by (OVA)S**^**−/+**^**(CpG) or S**^**−/+**^**(OVA−CpG) *in vivo*. (A)** Determination of the antigen−specific killing using *in vivo* CTL assay. C57BL/6 mice (n = 3–5) were immunized with the indicated treatments *via* footpad injection. Each treatment contained 6 μg of OVA. On day 7 following immunization, a 1:1 splenocytes mixture consisting of target cells pulsed with 200 nM SIINFEKL and labelled with 0.5 μM CFSE and unpulsed control cell labelled with 5 μM CFSE was intravenously administered to the control or immunized mice. Splenocytes were harvested, 18 h later, from the control or immunized mice and analyzed using flow cytometry analysis. Antigen−specific killing induced by each treatment was determined. Each dot represents killing of target cells by each mouse, the mean value for each treatment is shown as a horizontal bar. **(B)** Quantification of OVA−specific IgG. C57BL/6 mice (n = 3) were immunized with the indicated treatments, *via* footpad injection, each treatment contained 6 μg of OVA and CpG. On day 21 following injection, control or immunized mice sera were collected. The OVA−specific IgG, IgG1 or IgG2c were determined using ELISA. Data represent the mean value ± S.D. *P < 0.05, **P < 0.01, ***P < 0.001.

It was revealed that immunization with S^−/+^(OVA−CpG) induced a greater level of antigen−specific killing (47.7% ± 11.7) in contrast to OVA−CpG (10.8% ± 4.2) or (OVA)S^−/+^(CpG) (29.1% ± 3.0) (P < 0.0001) (Fig. 3A and Figure S5).

To assess the humoral response induced by the conjugates *in vivo*, C57BL/6 mice were treated with the conjugates or appropriate controls, and OVA−specific antibodies were quantified using an OVA−specific ELISA 21 days post immunization (Fig. 3B). Immunization with S^−/+^(OVA−CpG) significantly boosted the production of anti−OVA IgG and IgG2c antibodies titres compared to (OVA)S^−/+^(CpG) or other treatments. Both conjugates, however, ensued comparable anti−OVA IgG1 titres.

These findings demonstrated further the augmentation in antigen specific immune response *in vivo* achieved by S^−/+^(OVA−CpG) over (OVA)S^−/+^(CpG). We adopted this conjugate S^−/+^(OVA−CpG) for subsequent studies in combination with αCD40 as a second immunoadjuvant.

### 3.4 Incorporation of αCD40 as a second immunoadjuvant improves OVA presentation *in vitro* and intensifies OVA−specific immune response *in vivo* even at lower OVA doses

**Fig. 4:**
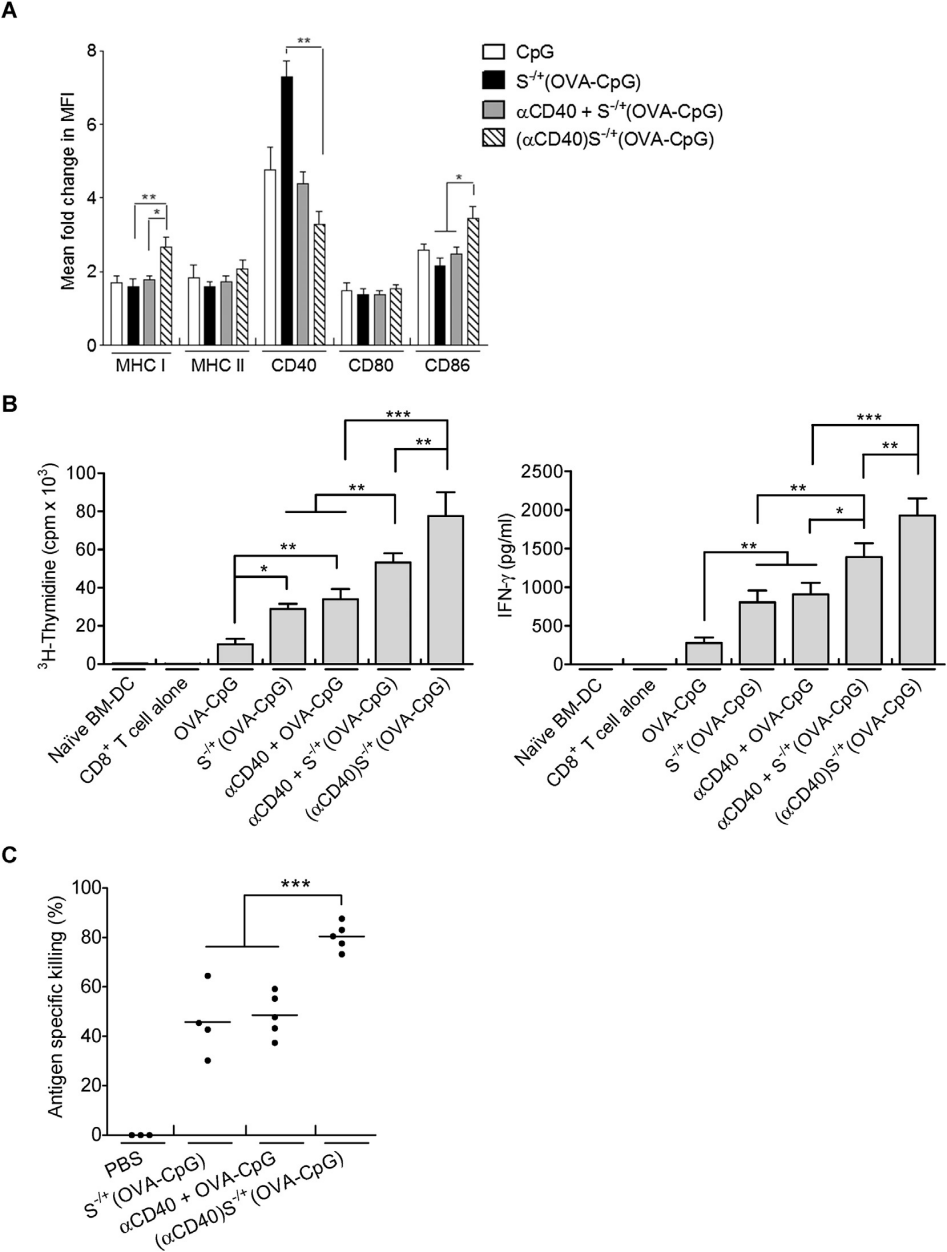
**Assessment of immune response induced by (αCD40)S**^**−/+**^**(OVA−CpG) *in vitro* and *in vivo*. (A)** Assessment of BM−DC maturation. BM−DCs were incubated for 24 h with S^−/+^(OVA−CpG), αCD40 + S^−/+^(OVA−CpG) or (αCD40)S^−/+^(OVA−CpG)) each contained 0.5 μg/ml OVA, 0.5 μg/ml CpG and/or 1.8 μg/ml αCD40. BM−DCs were stained with fluorescently labelled antibodies and analyzed using flow cytometry. The MFI was measured to assess the fold change in the expression of each marker with respect to naïve BM−DCs. **(B)** Assessment of OVA presentation. BM−DCs were incubated for 24 h with either 1 μg/ml OVA (contained in OVA−CpG or S^−/+^(OVA−CpG)) or 0.5 μg/ml OVA (contained in OVA−CpG + αCD40, S^−/+^(OVA−CpG) + αCD40 or (αCD40)S^−/+^(OVA−CpG)). S^−/+^ unconjugated or conjugated αCD40 was used at 1.8 μg/ml. BM−DCs were co−cultured with CD8^+^ T cells. (Left) CD8^+^ T cell proliferation. CD8^+^ T were pulsed with ^3^H−thymidine and proliferation was measured. (Right) IFN−γ quantification. The content of IFN−γ in the supernatants of the proliferating CD8^+^ T cell was quantified using ELISA. Measurements were performed in triplicates for each condition, results represent the mean ± S.D. **(C)** CTL response. C57BL/6 mice (n = 3–5) were immunized, *via* footpad injection, with either 6 μg OVA (contained in S^−/+^(OVA−CpG)) or 3 μg OVA (contained in OVA−CpG + αCD40 or (αCD40)S^−/+^(OVA−CpG)). The S^−/+^ unconjugated or conjugated αCD40 was 10 μg. Each dot represents killing of target cells by each mouse, the mean value for each treatment is shown as a horizontal bar. *P < 0.05, **P < 0.01, ***P < 0.001.

To further intensify the antigen−specific immune responses observed, αCD40 antibody was loaded onto S^−/+^ as a second immunoadjuvant. In order to assess the effect of αCD40 contained in (αCD40)S^−/+^(OVA−CpG) on DC activation markers, BM−DCs were stimulated with the conjugate or control treatments and known DC markers were assessed using flow cytometry as described in Supplementary Information. BM−DC stimulated with (αCD40)S^−/+^(OVA−CpG) expressed significantly higher levels of MHC I and CD86 compared to those stimulated with S^−/+^(OVA−CpG) or a mixture of unconjugated αCD40 and S^−/+^(OVA−CpG) (αCD40 + S^−/+^(OVA−CpG)) (Fig. 4A). BM−DCs stimulated with (αCD40)S^−/+^(OVA−CpG) showed lower expression of CD40 compared to S^−/+^(OVA−CpG). This could be attributed to the cellular internalization of the CD40 receptor following its ligation with αCD40 contained in (αCD40)S^−/+^(OVA−CpG) [20], [21].

Given the increase in MHC I expression, OVA presentation to CD8^+^ T cell was assessed, following stimulation with (αCD40)S^−/+^(OVA−CpG). In order to assess the synergy provided by the CpG and αCD40 loaded onto S^−/+^, BM−DCs were incubated with (αCD40)S^−/+^(OVA−CpG) containing half the OVA and CpG amounts present in S^−/+^(OVA−CpG) treatment (Fig. 4B). Higher CD8^+^ T cell proliferation was induced following stimulation with (αCD40)S^−/+^(OVA−CpG) containing 0.5 μg/ml of both OVA and CpG compared to the control treatment αCD40 + S^−/+^(OVA−CpG), and S^−/+^(OVA−CpG) treatment that contained 1 μg/ml of both OVA and CpG. Production of IFN−γ by the stimulated CD8^+^ T cells correlated well with their pattern of proliferation. These observations reflected the significance of αCD40 conjugation with S^−/+^ on BM−DC activation.

Immune enhancement induced by (αCD40)S^−/+^(OVA−CpG) was investigated using the *in vivo* CTL assay. Immunization of C57BL/6 mice with (αCD40)S^−/+^(OVA−CpG) (containing 3 μg OVA and CpG) led to a robust OVA−specific cellular immune response (80.4% ± 5.4 antigen−specific killing) (P < 0.0001) compared to the CTL response induced by the control αCD40 + OVA−CpG (containing 3 μg OVA and CpG) or S^−/+^(OVA−CpG) treatment (containing 6 μg OVA and CpG) showing 48.6% ± 8.8 or 46.2% ± 14.1 antigen−specific killing, respectively (Fig. 4C and Figure S5).

Taking the *in vitro* and *in vivo* data together we can conclude that inclusion of αCD40 antibody as a second immunoadjuvant resulted in synergy of the MWNT-mediated delivery of OVA−CpG as shown by the marked increase in antigen−specific immune responses at lower OVA and CpG doses.

### 3.5 αCD40 and OVA−CpG loading onto S^−/+^ effectively delays the tumour growth in both solid and lung pseudo−metastatic tumour models

The therapeutic efficacy of the conjugates in delaying the growth of a solid tumour was investigated. Immunization of C57BL/6 mice subcutaneously inoculated with Luc−B16F10−OVA cells with S^−/+^(OVA−CpG) containing 12 or 25 μg of both OVA and CpG led to significant tumour growth retardation compared to unimmunized mice (Figure S6A). Furthermore, administration of S^−/+^(OVA−CpG), containing 25 μg of both OVA and CpG, to mice subcutaneously inoculated with B16 cells (tumour cells which do not express OVA), failed to impede the B16 cells growth (Figure S6B), indicating that the induced anti−tumour immune response was antigen−specific.

**Fig. 5:**
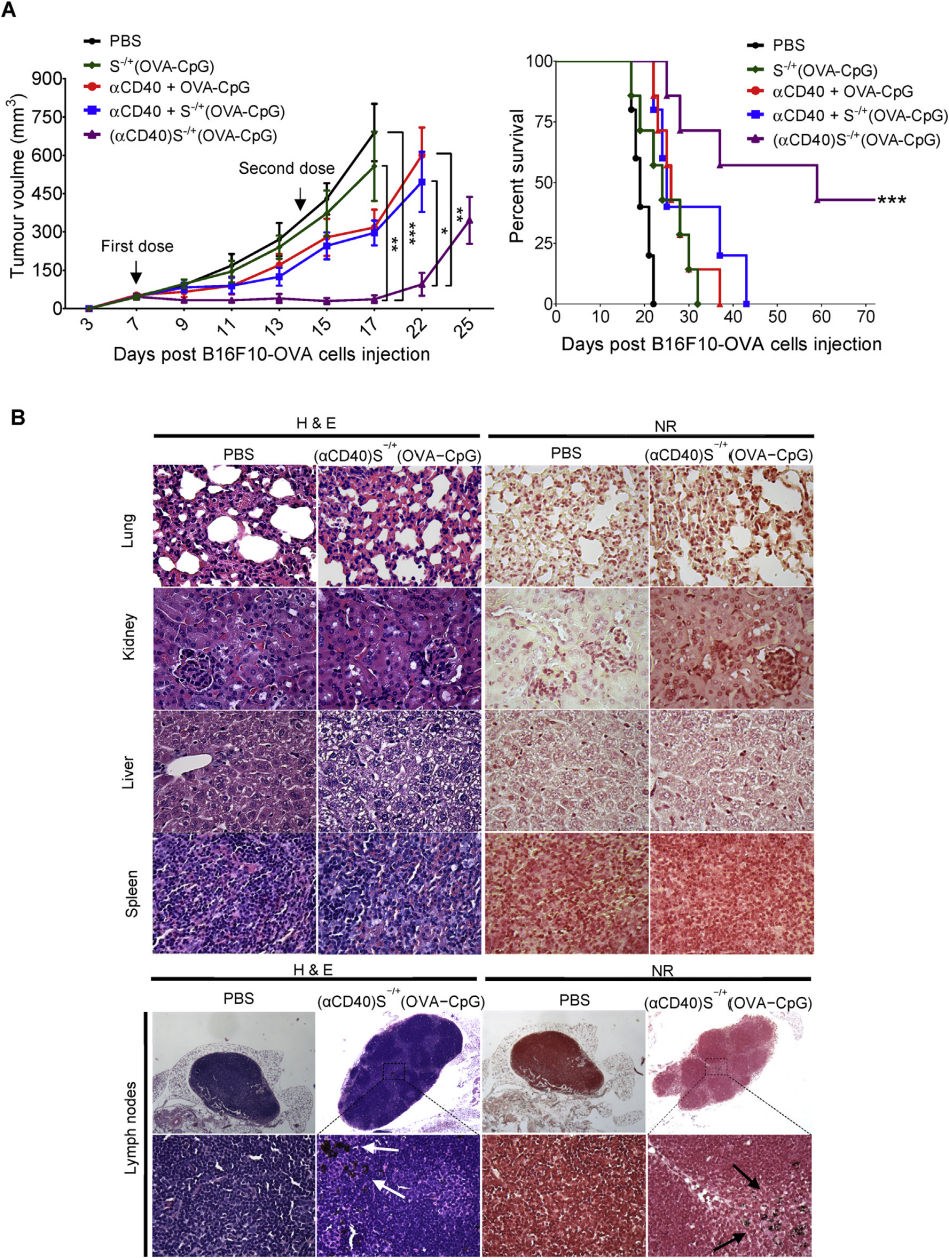
**Assessment of anti−tumour response in subcutaneous tumour models**. C57BL/6 mice (n = 7) were subcutaneously injected with 2.5 × 10^5^ OVA−B16F10−Luc cells. On the 7th and 14th days post tumour cells injection, tumour−inoculated mice were immunized *via* footpad injection with the indicated treatments, each contained 6 μg OVA. **(A)** Tumour growth curve and mice survival. (Left) Tumour growth monitored by calliper measurement. Values are expressed as mean value ± SEM. (Right) Tumour−inoculated mice survival. *P < 0.05, **P < 0.01, ***P < 0.001. **(B)** Histological analysis. The main organs and lymph nodes excised from scarified subcutaneous tumour inoculated mice stained with haematoxylin and eosin (H & E) (left) or neutral red (NR) (right). Images were captured at ×40 magnification. S^−/+^ appeared as dark black aggregates (arrows). (For interpretation of the references to colour in this figure legend, the reader is referred to the web version of this article.)

To determine the ability of (αCD40)S^−/+^(OVA−CpG) to delay the solid tumour growth at reduced OVA and CpG doses, mice were subcutaneously inoculated with Luc−B16F10−OVA cells and then immunized with (αCD40)S^−/+^(OVA−CpG), S^−/+^(OVA−CpG) or other controls at 6 μg of both OVA and CpG. As demonstrated in Fig. 5A, immunization with S^−/+^(OVA−CpG) at 6 μg OVA did not significantly delay the tumour growth compared to unimmunized mice. However, immunization with (αCD40)S^−/+^(OVA−CpG) led to significant tumour growth retardation compared to unimmunized mice, and mice immunized with S^−/+^(OVA−CpG). Immunization with αCD40 + S^−/+^(OVA−CpG) failed to delay the tumour growth to the same extent as (αCD40)S^−/+^(OVA−CpG), highlighting the increase in αCD40−mediated immune enhancement achieved on incorporating αCD40 onto S^−/+^ in addition to OVA−CpG. Additionally, vaccination with (αCD40)S^−/+^(OVA−CpG) prolonged the tumour−inoculated mice survival in a significant manner compared to the other treatments (Fig. 5A).

No changes in the histological features of the excised organs between the untreated and treated tumour bearing mice were observed indicating lack of organotoxicity (Fig. 5B). Dark black aggregates, which were absent in naïve mice, were detected in the popliteal lymph nodes from mice immunized with (αCD40)S^−/+^(OVA−CpG), suggesting drainage of S^−/+^ into the popliteal lymph nodes.

**Fig. 6:**
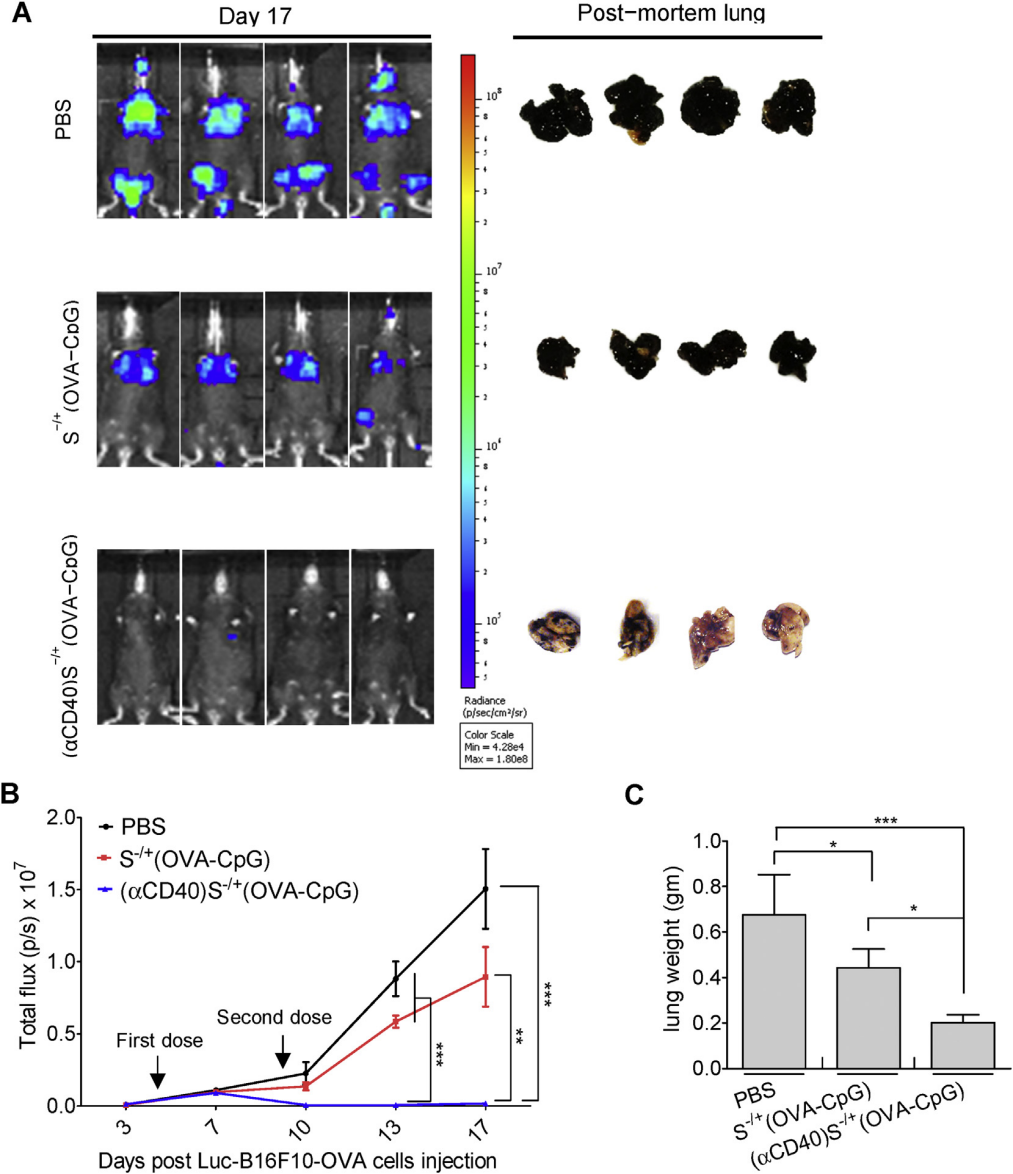
**Assessment of anti−tumour response in lung pseudo−metastatic tumour models**. C57BL/6 mice (n = 6–8) were intravenously injected with 2.5 × 10^5^ OVA−B16F10−Luc cells. On the 4th and 9th days post tumour cells injection, tumour−inoculated mice were immunized *via* footpad injection with the indicated treatments, each contained 6 μg OVA. **(A)** Lung pseudo−metastatic tumour model. Tumour growth was monitored by whole body imaging. Representative images for *in vivo* bioluminescent imaging and the corresponding post−mortem lung photographs are shown. **(B)** Quantification of photon flux, expressed as number of photons per second (p/s). Values are expressed as mean value ± SEM. **(C)** The weights of the lung excised from scarified tumour inoculated mice. Values are expressed as mean value ± S.D. *P < 0.05, **P < 0.01, ***P < 0.001.

Therapy studies were then performed in the more challenging pseudo−metastatic lung tumour model. The conjugates, containing 6 μg of both OVA and CpG, were administered to C57BL/6 mice previously intravenously inoculated with OVA−B16F10−Luc cells. Smaller bioluminescence signals and lung weights were observed in mice immunized with (αCD40)S^−/+^(OVA−CpG) compared to S^−/+^(OVA−CpG) treated mice (Fig. 6).

From our data we conclude that vaccination with (αCD40)S^−/+^(OVA−CpG) efficiently delayed the OVA−B16F10−Luc tumour growth in both solid and pseudo−metastatic tumour models, consistent with the immune enhancements observed *in vitro* and *in vivo*.

## 4 Discussion

One of the purposes of using delivery vectors for antigens is to improve the antigen uptake by the antigen presenting cells (APCs) in order to increase the intracellular antigen concentration, thus the density of antigen presented by the APCs to T cells. It has been previously reported that polymeric spherical nanoparticles, e.g. PLGA nanoparticles, mainly utilise energy−dependent mechanisms of cellular uptake rather than energy−independent ones [36], [37], [38]. The reported findings that demonstrated the CNTs' ability to enter the cells *via* more than one route i.e. energy−dependent and/or passive routes [4], [5], [6], may suggest that CNTs can deliver higher amounts of antigens into the APCs compared to spherical nanoparticles. However, comparative studies need to be carried out to investigate the cellular uptake of CNTs versus the extensively studied spherical nanoparticles, e.g. PLGA nanoparticles and liposomes, by the APCs and the ensuing effects on the magnitude of immune response elicited against incorporated antigen.

Covalent conjugation of OVA and CpG and their loading onto S^−/+^ improved OVA presentation *in vitro* by BM−DCs and efficiently elevated the magnitude of OVA−specific immune response *in vivo*. Additionally, the presence of αCD40 in S^−/+^ containing conjugated OVA−CpG led to i) more advanced augmentation of the OVA−specific immune response *in vitro* and *in vivo*, and ii) delayed growth of OVA−expressing B16F10 cells effectively in both subcutaneous and lung pseudo−metastatic tumour models, at reduced OVA and CpG doses.

We initially proposed two distinct approaches for the concomitant delivery of the model antigen OVA and CpG using S^−/+^ to APCs. Mixing S^−/+^ with OVA and CpG was the first method, yielding (OVA)S^−/+^(CpG); however, the loading of OVA and CpG onto each S^−/+^ was uncontrolled. In other words, the prepared (OVA)S^−/+^(CpG) might possessed lower OVA and CpG co−loading onto each S^−/+^ compared to S^−/+^(OVA−CpG), and the formation of OVA or CpG only −conjugated S^−/+^ was also possible. Accordingly, the other approach was loading both agents in the form of the chemical conjugate, OVA−CpG, to ensure the co−incorporation of OVA and CpG onto the same S^−/+^ (S^−/+^(OVA−CpG)). PAGE gel results confirmed that OVA contained in S^−/+^(OVA−CpG) was in the CpG−conjugated form. (OVA)S^−/+^(CpG) or S^−/+^(OVA−CpG) elicited higher immune response potency *in vitro* and *in vivo,* compared to their control treatments, namely the mixture of unconjugated OVA and CpG or OVA−CpG, respectively. This was in agreement with the previously reported benefits of CNTs as a delivery vehicle for antigens [7], [9] or immunoadjuvants [11], [12] *in vitro* and *in vivo*.

The co−delivery of antigen and CpG to APC has also been demonstrated using other particulate delivery systems. For instance, mice immunization with microparticles co−encapsulating OVA and CpG increased the anti−OVA antibodies [39] and CD8^+^ T cell responses [40], [41], and utilization of gold nanoclusters for the co−delivery of OVA−derived peptide and CpG augmented the production of anti−OVA antibodies in mice [42].

In a study by de Faria et al., OVA and CpG were co−incorporated onto MWNTs by mixing MWNTs with un−conjugated OVA and CpG, yielding a conjugate that induced higher immune response compared to mixture of unconjugated OVA and CpG (MWNT-free) *in vivo* [43]. We utilized the same approach to prepare (OVA)S^−/+^(CpG), but additionally, we introduced in this study a more robust approach for comparison, where a covalently conjugated OVA and CpG were loaded onto MWNTs yielding S^−/+^(OVA−CpG). When, comparing the two MWNT based conjugates for OVA and CpG co−delivery, S^−/+^(OVA−CpG) resulted in better OVA presentation by BM−DCs than (OVA)S^−/+^(CpG). This could be attributed to the better capability of S^−/+^(OVA−CpG), compared to (OVA)S^−/+^(CpG), to co−internalize OVA and CpG into the same BM−DC. The enhanced OVA presentation might account for the higher cellular and humoral immune responses elicited by vaccination of C57BL/6 mice with S^−/+^(OVA−CpG). Similar to our findings, but without the use of a delivery system, previous studies have demonstrated that mice immunization with covalently conjugated OVA and CpG induced higher OVA−specific immune response compared to immunization with a mixture of unconjugated OVA and CpG [15], [28].

Schlosser et al. demonstrated that mixing PLGA polymer with OVA and CpG yielded microparticles that were described as OVA and CpG co−encapsulating microparticles, these microparticles induced higher CD8^+^ T cell response *in vitro* and *in vivo* in contrast to a mixture of OVA only−encapsulating microparticle and CpG only−encapsulating microparticle [44]. Similarly, Li et al. mixed lipid polymer with HER−2/neu derived peptide and CpG to yield liposomes that were referred to as antigen and CpG co−encapsulating liposomes, mice immunization with these liposomes induced higher immune response compared to a mixture of antigen−containing liposome and CpG−containing liposome [45]. The approach applied in these studies for antigen and CpG co−incorporation into a delivery system by mixing polymers with unconjugated antigen and CpG is similar, in its basic principle, to the one we followed for the synthesis of (OVA)S^−/+^(CpG) but not S^−/+^(OVA−CpG). These studies highlighted the importance of antigen and CpG co−delivery using a delivery system by comparing co−incorporated to separately incorporated antigen and CpG. However, our study is introducing a more advanced line of complexity by contrasting two methods for antigen and CpG concomitant delivery using a delivery vehicle as demonstrated by (OVA)S^−/+^(CpG) versus S^−/+^(OVA−CpG).

Previous studies reported the use of spherical−shaped delivery systems to improve antigen and αCD40 co−delivery. Hatzifoti et al. demonstrated that co−encapsulation of tetanus toxoid and αCD40 in liposomes augmented the antigen−specific antibody response in BALB/c mice [46], and Rosalia et al. reported an increase in CD8^+^ T cell response following mice immunization with αCD40−coated PLGA nanoparticles co−incorporating an oncoprotein and ligands for TLR2 and TLR3 [47]. Instead of harnessing the conventional spherical particulate delivery systems to co−deliver CpG and αCD40, we utilized an emerging cylindrical vectors, namely the MWNTs, as a nano−carrier for both CpG and αCD40. In addition, the therapeutic outcome provided by the MWNT-delivered CpG and αCD40 was not only evaluated in a standard subcutaneous tumour model but also lung pseudo-metastatic tumour model.

The intensified strength of OVA specific−CTL response induced by vaccination of C57BL/6 mice with (αCD40)S^−/+^(OVA−CpG) might be assigned to the better ability of this conjugate to induce DC maturation and to further fortify OVA presentation as observed *in vitro*. Stimulation of APC with αCD40 has been shown to upregulate MHC I and CD86 expression [48], [49]. Intracellular signalling induced by ligation of CD40 receptor with αCD40 requires CD40 receptor cross−linking that increases, accordingly, with the increase in the number of αCD40 interacting at the cell surface [50]. The observed better ability of (αCD40)S^−/+^(OVA−CpG) than αCD40 + S^−/+^(OVA−CpG) in upregulating MHC I and CD86 expression by BM−DCs could be attributed to the more efficient CD40 receptor cross−linking by the multiple, surface−bound, αCD40 contained in (αCD40)S^−/+^(OVA−CpG) [51]. Expression of CD86 by APCs was increased following stimulation with αCD40−coated polymeric nanoparticles [51] or silicon nanoparticles [52] compared to free αCD40.

The fact that the MWNT−based conjugates were detected in the lymph nodes was in agreement with our previous study. Where, we were able to detect the presence of S^−/+^ and the processing of S^−/+^ conjugated OVA in the CD11c^+ve^ DCs subsets in the popliteal lymph nodes [7]. These observations reflected the proficiency of MWNT as vaccine delivery vectors. Since efficient antitumour−immune response induction demands antigen trafficking through the lymphatic vessels and internalization by the lymph node−residing CD8^+^ DC, which is the only DC subset capable of inducing CD8^+^ T cell response *in viv*o [53].

Efficient eradication of B16−OVA or B16F10−OVA tumours in mice has been found to be associated with the cytolytic activity of CD8^+^ T cells demonstrated by OVA−specific CTL response [41], [47], [54]. The fact that lower OVA and CpG doses were required by (αCD40)S^−/+^(OVA−CpG) than S^−/+^(OVA−CpG) to induce strong anti−tumour response indicates the higher potency and better efficacy of the former conjugate *in vivo*. Collectively, the results shown in this study highlighted the exploitation of MWNTs as antigen and immunoadjuvants nanocarrier for the purpose of inducing potent anti−tumour immune response.

## 5 Conclusions

OVA incorporation onto the MWNT in the form of CpG−conjugated OVA improved the CpG−mediated enhancements of OVA−specific immune response *in vitro* and *in vivo*. Furthermore, the utilization of MWNTs as vaccine delivery vector has intensified the CpG and αCD40−derived synergism that markedly retarded the OVA−B16F10 growth in the tested tumour models. The MWNT−delivered immuno−based combinatorial therapeutic approach presented in this study could be exploited for potent anti−tumour immune response induction against challenging cancer diseases.

## Figures and Tables

**Table 1:** Physicochemical characterization of conjugates.

	Length^a^^,^^b^ (nm)	Primary amine loading^b^^,^^c^ (μmole/g S^−/+^)	OVA loading^b^^,^^d^ (mg/g S^−/+^) [μmol/g S^−/+^]	CpG loading^b^^,^^e^ (mg/g S^−/+^) [μmol/g S^−/+^]	αCD40 loading^b^^,^^d^ (mg/g S^−/+^) [μmol/g S^−/+^]
(OVA)S^−/+^(CpG)	122 ± 82	263 ± 72	205 ± 24 [4.5 ± 0.53]	288 ± 20 [45 ± 3.1]	–
S^−/+^(OVA−CpG)	122 ± 82	263 ± 72	130 ± 21 [2.9 ± 0.47]	136 ± 18 [21.2 ± 2.8]	–
(αCD40)S^−/+^(OVA−CpG)	122 ± 82	263 ± 72	55 ± 15 [1.2 ± 0.33]	57 ± 11 [8.8 ± 1.7]	188 ± 17 [1.3 ± 0.1]

a Determined from TEM images (n = 100 nanotubes).

b Data are represented as mean ± SD.

c Measured using TGA (n = 3).

d Measured using BCA assay (n = 3).

e Measured using NanoDrop (n = 3).
