# Supplementary material for: Dual stimulation of antigen presenting cells using carbon nanotube-based vaccine delivery system for cancer immunotherapy
Source: Biomaterials. 2016 Oct;104:310–22. doi: 10.1016/j.biomaterials.2016.07.005 (PMC4993816; doi:10.1016/j.biomaterials.2016.07.005)
Supplement: Supplementary file 1 [file mmc1.pdf]

Dual stimulation of antigen presenting cells using carbon nanotube−based vaccine delivery system for cancer immunotherapy.

Hatem A. F. M. Hassan[1]*, Lesley Smyth*[2]*, Julie T.−W. Wang*[1]*, Pedro M. Costa*[1]*, Kulachelvy Ratnasothy*[2]*, Sandra S. Diebold*[3]*, Giovanna Lombardi*[2*]*, Khuloud T. Al−Jamal*[1*]

Hatem A. F. M. Hassan, Pedro M. Costa, Julie T.−W. Wang, Khuloud T. Al−Jamal

[1]Institute of Pharmaceutical Science, Faculty of Life Sciences & Medicine, King's College London, Franklin−Wilkins Building, London SE1 9NH, United Kingdom

Lesley Smyth, Kulachelvy Ratnasothy, Giovanna Lombardi

[2]Immunoregulation Laboratory, MRC Center for Transplantation, King’s College London, Guy’s Hospital, London SE1 9RT, United Kingdom

Sandra S. Diebold

[3]Division of Immunology, Infection, and Inflammatory Diseases, King’s College London, Guy’s Hospital, London SE1 9RT, United Kingdom

*Corresponding authors: khuloud.al-jamal@kcl.ac.uk, giovanna.lombardi@kcl.ac.uk

Current address for Lesley Smyth:

School of Health, Sport and Biosciences, University of East London, Stratford Campus, Water Lane, London E15 4LZ, UK

Current address for Sandra S. Diebold:

Immunotoxicology Team, Biotherapeutics Division, National Institute for Biological Standards and Control (NIBSC), Blanche Lane, South Mimms, Potters Bar, Hertfordshire, EN6 3QG, UK

Supplementary Information

Supplementary materials

Ethylenediaminetetraacetic acid (EDTA) was purchased from Sigma (UK). Bicinchoninic acid (BCA) protein assay reagent was purchased from Fisher Scientific (UK). Sulfo−SMCC was purchased from Biovision (USA). Amicon Ultra 0.5 ml 30 k and Amicon Ultra 0.5ml 3 k were purchased from Millipore (Germany). Horseradish peroxidise (HRP) conjugated goat anti-mouse IgG was purchased from Cayman chemical (USA). HRP conjugated goat anti-mouse IgG1 was purchased from Bethyl laboratories (USA). HRP conjugated goat anti-mouse IgG2c was purchased from Southern Biotech (USA). Penicillin, streptomycin, RPMI 1640 medium, 2-mercaptoethanol, L-glutamine, HEPES buffer solution and SYBR Gold were purchased from Life Technologies (UK). 10% heat inactivated foetal calf serum (FCS) was purchased from First Link (UK). Allophycocyanin (APC) anti−CD11c, fluorescein isothiocyanate (FITC) anti−H−2kb, phycoerythrin (PE) anti−I−A[b], PE CD40, PE anti−CD80, PE anti−CD86, FITC IgG2a, κ isotype, PE IgG2a κ isotype, PE IgG2a κ isotype, PE IgG2 κ isotype, FITC IgG2b κ isotype, APC IgG2a κ isotype were purchased from Becton Dickinson (USA).

Supplementary methods

Synthesis of OVA−CpG conjugate

Synthetic steps are illustrated in **Scheme S1**.

Maleimide−activation of OVA

OVA was dissolved in PBS at 10 mg/ml. Sulfo−SMCC was dissolved in de−ionized water at 5 mg/ml. To 165 µl of a reaction buffer consisting of 0.1 M NaCl in PBS (pH 6.9), 135 µl of OVA solution (1.35 mg OVA) and 75 µl of sulfo−SMCC solution were transferred. The reactants were stirred together for 90 min at RT. Using the 0.1 M NaCl/PBS buffer and a 30 kD Amicon 0.5 spin filter, unreacted sulfo−SMCC was removed by spinning the 30 kD Amicon 0.5 spin filter at 14000 rpm for 12 min. The washing step was repeated three more times; the last washing step was performed using a conjugation buffer consisting of 2 mM EDTA in PBS (pH 7.1). Maleimide−activated OVA was recovered from the 30 kD Amicon 0.5 spin filter as a concentrated solution of 20−30 µl.

Activation of thiol−modified CpG

Thiol−modified CpG was dissolved in PBS at 10 mg/ml. DTT was dissolved in water at 77 mg/ml. To 200 µl of thiol−modified CpG (2 mg CpG), 20 µl of DTT were added and stirred together for 60 min at RT. Using the 0.1 M NaCl/PBS buffer and a 3 kDa Amicon 0.5 spin filter, excess DTT was removed by spinning at 14000 rpm for 12 min. The washing step was repeated three more times; the last washing step was performed using a conjugation buffer consisting of 2 mM EDTA in PBS (pH 7.1). Activated thiol−modified CpG was recovered from the 3 kDa Amicon 0.5 spin filter as a concentrated solution of 30−40 µl.

OVA conjugation with CpG

Maleimide−activated OVA and activated thiol−modified CpG were mixed and stirred together for 90 min at RT. Using the 0.1 M NaCl/PBS buffer and a 3 kDa Amicon 0.5 spin filter, excess CpG was removed by spinning at 14000 rpm for 12 min. The washing step was repeated six more times; the last washing step was performed using PBS. OVA−CpG conjugate was recovered from the 3 kDa Amicon 0.5 spin filter as a concentrated solution of 70 µl. Conjugated OVA and CpG content was determined using BCA assay and NanoDrop, respectively.

Determination of OVA content in synthesized conjugates using BCA assay

The BCA assay was performed as previously described with some modifications [1]. A calibration curve was prepared using 25−2000 µg/ml of OVA in PBS (pH7.4). For the quantification of OVA contained in the OVA−CpG conjugate, collected samples were diluted 25 times prior measurements. Following the determination of unreacted OVA concentration in the collected filtrates, OVA content in $S^{-/+}$(OVA−CpG), (OVA)$S^{-/+}$(CpG) or (αCD40)$S^{-/+}$(OVA−CpG) was calculated using the following equation as µg of OVA per mg of $S^{-/+}$:

$$\frac{[\text{Initially added amount of OVA }(\mu\text{g}) - \text{ unreacted OVA detected in the filterate }(\mu\text{g})]}{[\text{Initial weight of } \text{S}^{-/+}(\text{mg}) \text{ added to the reaction}]}$$

Determination of CpG content in synthesized conjugates using NanoDrop

To determine the CpG content in OVA−CpG conjugate, standard concentrations of CpG were prepared ranging from 20 to 800 µg/ml, each standard was spiked with OVA at a final concentration of 500 µg/ml. The concentration of the prepared CpG standards was measured using NanoDrop ND−1000 spectrophotometer (NanoDrop Technologies, Wilmington, USA). A calibration curve was plotted for the measured CpG concentration versus the prepared CpG concentration. The OVA content in the OVA−CpG sample was adjusted to 500 µg/ml, afterwards CpG concentration was measured using NanoDrop. From the calibration curve the actual CpG concentration was determined. The same procedure was followed for the determination of CpG content in $S^{-/+}$(OVA−CpG), (OVA)$S^{-/+}$(CpG) or (αCD40)$S^{-/+}$(OVA−CpG) with few modifications. Standard concentrations of CpG were prepared ranging from 20 to 800 µg/ml, each standard was spiked with OVA at a final concentration of 150 µg/ml. OVA concentration in filtrate samples collected following filtration of $S^{-/+}$(OVA−CpG), (OVA)$S^{-/+}$(CpG) or (αCD40)$S^{-/+}$(OVA−CpG) was adjusted to 150 µg/ml before determining the CpG concentration. Following the determination of unreacted CpG concentration in the collected filtrates, CpG content in $S^{-/+}$(OVA−CpG), (OVA)$S^{-/+}$(CpG) or (αCD40)$S^{-/+}$(OVA−CpG) was calculated using the following equation as µg of CpG per mg of $S^{-/+}$:

$$\frac{[\text{Initial amount of CpG } (\mu\text{g}) - \text{ unreacted CpG detected in the filterate } (\mu\text{g})]}{[\text{Initial weight of } \text{S}^{-/+} \text{ (mg) added to the reaction}]}$$

Determination of αCD40 content in (αCD40)$S^{-/+}$ using BCA assay

A calibration curve was prepared using 25−500 µg/ml of αCD40 in PBS and the BCA assay was carried out as described before. αCD40 content in (αCD40)$S^{-/+}$ was calculated using the following equation as µg of αCD40 per mg of $S^{-/+}$:

$$\frac{[\text{Initial amount of } \alpha\text{CD40 } (\mu\text{g}) - \alpha\text{CD40 detected in the filterate } (\mu\text{g})]}{[\text{Initial weight of } \text{S}^{-/+} \text{ (mg) added to the reaction]}}$$

***In vitro* stability**

$S^{-/+}$(OVA) and $S^{-/+}$(CpG) were dispersed in PBS (pH 7.4) at 1 mg/ml and transferred to dialysis membranes of 100 kDa MWCO and 10 kDa MWCO, respectively. The dialysis membranes were then exposed to constant agitation in PBS (pH 7.4) at 37 °C for 1, 3 or 7 days. To determine OVA or CpG contained in the conjugates at the specified time points by gel electrophoresis, $S^{-/+}$(OVA) containing 10 µg OVA was transferred to the wells of 15% native, non-reducing gel and $S^{-/+}$(CpG) containing 1 µg CpG was transferred to the wells of 2% agarose gel. OVA and CpG bands were detected by gel staining with Coomassie Brilliant blue and SYBR Gold, respectively.

Culture medium

The complete culture medium used throughout the experiments consisted of RPMI 1640 medium, supplemented with 100 IU/ml penicillin, 100 µg/ml streptomycin, 2 mM L−glutamine, 50 µM 2−Mercaptoethanol, 0.01 M HEPES buffer solution and 10% heat inactivated FCS. Cells were incubated in a humidified atmosphere of 5% CO_2 at 37 ºC.

Determination of BM−DC phenotypes following treatment with (OVA)$S^{-/+}$(CpG) and $S^{-/+}$(OVA−CpG) *in vitro*

BM−DCs were incubated for 24 hr with OVA, CpG, (OVA)$S^{-/+}$(CpG) or $S^{-/+}$(OVA−CpG) each contained 5 µg/ml OVA. Expression of MHC I, MHC II, CD40, CD80 or CD86 by the CD11c^{+ve} BM−DCs was determined using flow cytometry as described before [1]. Briefly, BM−DCs harvested following the incubation period were incubated for 30 minutes with a mixture of fluorescently−labelled antibodies consisting of anti−CD11c antibody and anti−MHC I, MHC II, CD40, CD80 or CD86 antibody. BM−DCs incubated with fluorescently−labelled isotype non−specific antibodies were used as controls. Stained BM−DCs were analyzed using CellQuest software (BD Bioscience, US) operated FACSCalibur and subsequent analysis was carried out using FlowJo software (TreeStar, US).

Optimization of OVA/CpG doses required for induction of OVA presentation *in vitro*

BM−DCs were incubated for 24 h with OVA + CpG or OVA−CpG each contained 1, 5 or 10 µg/ml of both OVA and CpG. As a control, BM−DCs were incubated for 24 h with OVA alone 1, 5 or 10 µg/ml.

Treated BM−DCs were harvested and co−cultured with $CD8^+$ T cells isolated from OT−I mice spleen at 1:4 ratio and $CD8^+$ T cells proliferation was assessed using ^3H−thymidine incorporation assay.

Quantification of OVA−specific antibodies produced following immunization with (OVA)$S^{-/+}$(CpG) or $S^{-/+}$(OVA−CpG) in mice using ELISA

C57BL/6 mice (n= 3) were injected *via* the footpad with PBS, OVA−CpG, (OVA)$S^{-/+}$(CpG) or $S^{-/+}$(OVA−CpG) each at 6 μg OVA. At day 21 post injection, mice were bled *via* the inferior vena cava, collected blood samples were allowed to clot at R.T. then mice sera were separated by centrifugation at 16000 g for 20 min. Quantification of OVA−specific antibodies in the mice sera using ELISA was performed using a previously reported method [2]. Using 100 μl per well of 1 μg/ml OVA in PBS (pH 7.4), wells of high protein binding polystyrene 96−well plate (Corning Costar, USA) were coated and incubated overnight at 4 ^{o}C. The plate was 3 times washed using 0.05% Tween 20 in PBS and blocking was performed using 4% BSA in Tween 20/PBS for 1 h at R.T. The plate was 3 times washed and mice sera were two fold diluted using Tween 20/PBS then transferred in triplicates to the plate and incubated for 3 hr at R.T. Following the mice sera removal, the plate was five times washed using Tween 20/PBS. Horse radish peroxidise−conjugated goat anti−mouse IgG, IgG1 and IgG2c were diluted in Tween 20/PBS at 1:10,000 then 100 μl of each were transferred to the plate and incubated for 1 hr at R.T. The plate was five times washed using Tween 20/PBS. OVA−specific antibodies were detected by the addition of TMB, reaction was stopped by the addition of 0.2 M sulphuric acid. The absorbance was measured at 450 nm using (FLUOstar Omega, BMG LABTECH, Germany).

Determination of BM−DC phenotypes following treatment with (αCD40)$S^{-/+}$(OVA−CpG) *in vitro*

BM−DCs were incubated for 24 hr with $S^{-/+}$(OVA−CpG), mixture of unconjugated αCD40 and $S^{-/+}$(OVA−CpG) or (αCD40)$S^{-/+}$(OVA−CpG) each contained 0.5 μg/ml OVA, 0.5 μg/ml CpG and/or 1.8 μg/ml αCD40. As a positive control BM−DCs were treated with 2.5 μg/ml CpG. Expression of MHC I, MHC II, CD40, CD80 or CD86 by the $CD11c^{+ve}$ BM−DCs was determined using flow cytometry as described before.

Assessment of the anti−tumour response induced by $S^{-/+}$(OVA−CpG) in melanoma OVA−B16F10−Luc subcutaneous tumour model

C57BL/6 mice (n= 8) were subcutaneously inoculated in both flanks with 2.5×10^5 OVA−B16F10−Luc. On the 3rd and 10th days post tumour inoculation mice were immunized *via* footpad injection with $S^{-/+}$(OVA−CpG) containing 12 or 25 µg of both OVA and CpG in 50 µl PBS. PBS injected mice were used as untreated controls. A calliper was used to measure the tumour length (L) and width (W), and the tumour volume was calculated using the following equation: Tumour volume= $0.52 \times W^2 \times L$. Mice were sacrificed when the tumour volume reached 1000 mm^3.

Assessment of the anti−tumour response induced by $S^{-/+}$(OVA−CpG) in melanoma B16 subcutaneous tumour model

C57BL/6 mice (n= 8) were subcutaneously inoculated in both flanks with 2.5×10^5 B16 cells (obtained from Cancer Research UK Cell Service Laboratory). On the 3rd and 10th days post tumour inoculation, mice were immunized *via* footpad injection with $S^{-/+}$(OVA−CpG) containing 25 µg of both OVA and CpG in 50 µl PBS. PBS injected mice were used as untreated controls. Tumour growth was monitored using calliper measurements as described before.

Assessment of anti−tumour response induced by $S^{-/+}$(OVA−CpG) in melanoma OVA−B16F10−Luc lung pseudo−metastatic tumour model

C57BL/6 mice were intravenously inoculated with 2.5×10^5 OVA−B16F10−Luc cells. On the 7th day post tumour inoculation, mice were randomly assigned to 2 groups (n= 6−8). On the 4th and 9th days post tumour inoculation, mice were immunized *via* footpad injection with $S^{-/+}$(OVA−CpG) containing 12 µg of both OVA and CpG. PBS injected mice were used as untreated controls. Tumour growth was monitored by detecting the bioluminescence emitted from the inoculated OVA−B16F10−Luc cells following D−Luciferin injection. Every 3−4 days post tumour inoculation, mice were anesthetized and subcutaneously injected with D−Luciferin (150 mg/kg) in PBS. Imaging was performed using IVIS Lumina III and images analysis was conducted with Living Image® 4.3.1 Service Pack 2 software (Perkin Elmer, UK).

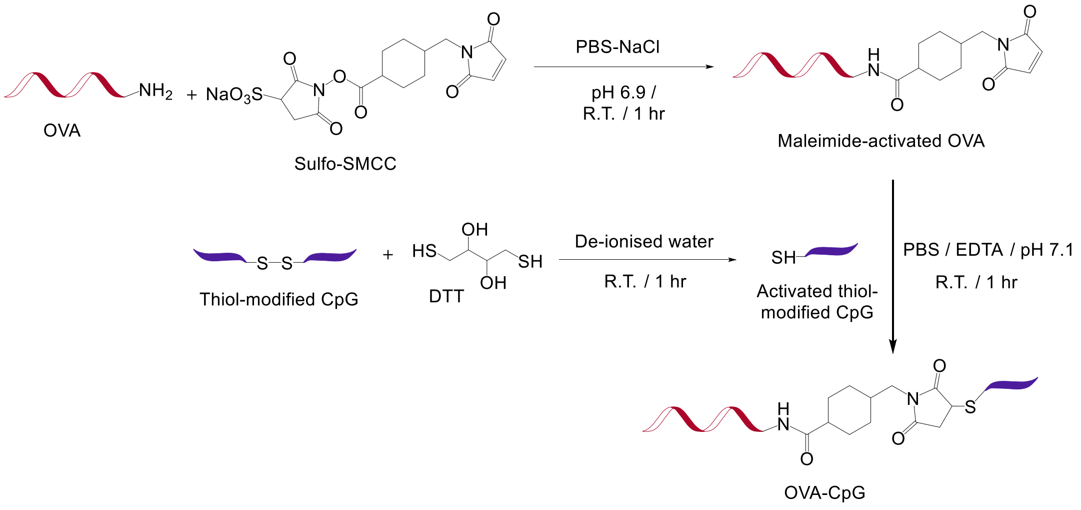

Scheme S1. Synthesis of OVA−CpG conjugate. OVA was maleimide−activated using sulfo−SMCC then covalently conjugated with the thiol−modified CpG *via* thioether linkage yielding OVA−CpG.

Table S1. Zeta potential of the functionalized MWNTs and synthesized conjugates.

	Zeta potential[a] (mV)
MWNT 1	−20.9
$S^{-/+}$	−7.27
(OVA)$S^{-/+}$(CpG)	−43.7
$S^{-/+}$(OVA−CpG)	−41.9

[a]Analyzed by electrophoretic mobility using 10× diluted PBS buffer.

Table S2. Loading efficiencies of the conjugates.[a]

	OVA loading efficiency (%)	CpG loading efficiency (%)	αCD40 loading efficiency (%)
(OVA)$S^{-/+}$(CpG)	41 ± 6.8	52.4 ± 5.2	−
$S^{-/+}$(OVA−CpG)	26 ± 5.9	24.8 ± 4.6	−
(αCD40)$S^{-/+}$(OVA−CpG)	16.5 ± 6.4	15.5 ± 4.2	56.4 ± 7.2

[a] Calculated as the percentage of OVA, CpG or αCD40 bound to $S^{-/+}$ of the starting material. Data are represented as mean ± SD (n= 3).

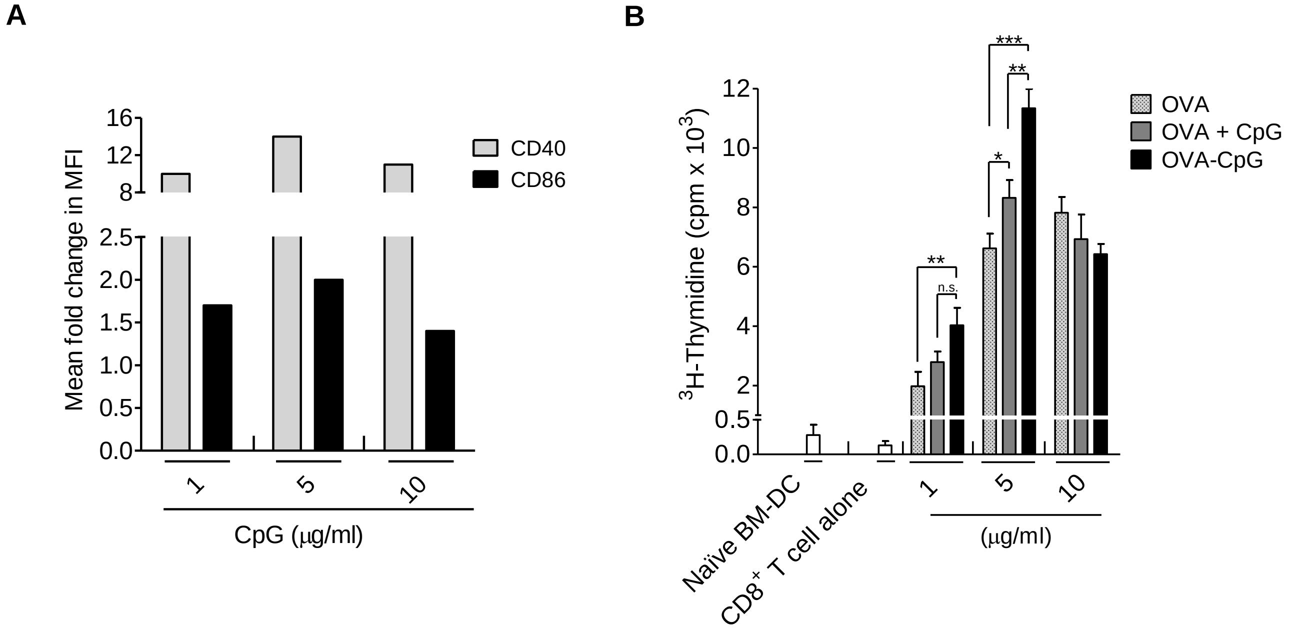

Figure S1. Optimization of CpG or OVA/CpG doses required for induction of BM−DC maturation or OVA presentation *in vitro*, respectively. (A) Dose−dependant stimulation of BM−DC maturation by CpG *in vitro*. BM−DCs were incubated for 24 h with 1, 5 or 10 µg/ml CpG and then analyzed for CD40 or CD86 expression using flow cytometry. BM−DCs were stained with specific fluorescently labelled antibodies or their corresponding isotype control antibodies, 10 x 10^4 cells were acquired using flow cytometry and the analysis was performed using FLowJo 7.6.5 software. The MFI of the positive CD11c−expressing BM−DCs was measured to assess the fold change in the expression of each marker with respect to the naïve BM−DCs. The optimal CpG dose for induction of BM−DC maturation was found to be 5 µg/ml. **(B)** Dose−dependant stimulation of $CD8^+$ T cell proliferation *in vitro* by OVA + CpG or OVA−CpG. BM−DCs were incubated for 24 h with OVA + CpG or OVA−CpG each contained 1, 5 or 10 µg/ml of both OVA and CpG. As a control, BM−DCs were incubated for 24 h with OVA alone at 1, 5 or 10 µg/ml. Treated BM−DCs were co−cultured with $CD8^+$ T cells, isolated from the spleen of OT−I C57BL/6 mice, at 1:4 ratio for 3 days. On the last 18 h, $CD8^+$ T were pulsed with 1 µCi of ^{3}H−thymidine and the $CD8^+$ T cell proliferation was measured using ^{3}H−thymidine incorporation assay. Measurements were performed in triplicates for each condition, results represent the mean ± S.D. * $P < 0.05$, ** $P < 0.01$, *** $P < 0.001$. Stimulation of BM−DCs with 5 µg/ml of both OVA and CpG contained in with OVA + CpG or OVA−CpG was found optimal for OVA presentation.

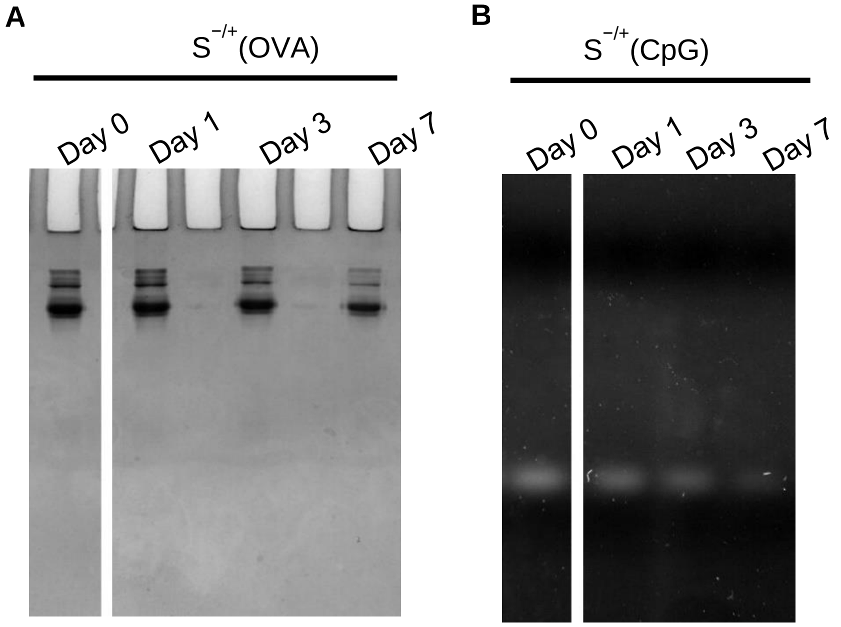

Figure S2. ***In vitro* stability. (A)** Assessment of *in vitro* stability of S$^{-/+}$(OVA) using PAGE. S$^{-/+}$(OVA) was dispersed in PBS (pH 7.4) at 1 mg/ml and exposed to constant agitation for 1, 3 or 7 days at 37 °C. At the indicated time points, S$^{-/+}$(OVA) containing 10 µg OVA was transferred to the wells of 15% native, non-reducing gel. Bands were detected by gel staining with Coomassie Brilliant blue. **(B)** Assessment of *in vitro* stability of S$^{-/+}$(CpG) using agarose gel electrophoresis. S$^{-/+}$(CpG) was dispersed in PBS (pH 7.4) at 1 mg/ml and exposed to constant agitation for 1, 3 or 7 days at 37 °C. At the indicated time points S$^{-/+}$(CpG) containing 1 µg CpG was transferred to the wells of a 2% agarose gel. Bands were detected by gel staining with SYBR Gold.

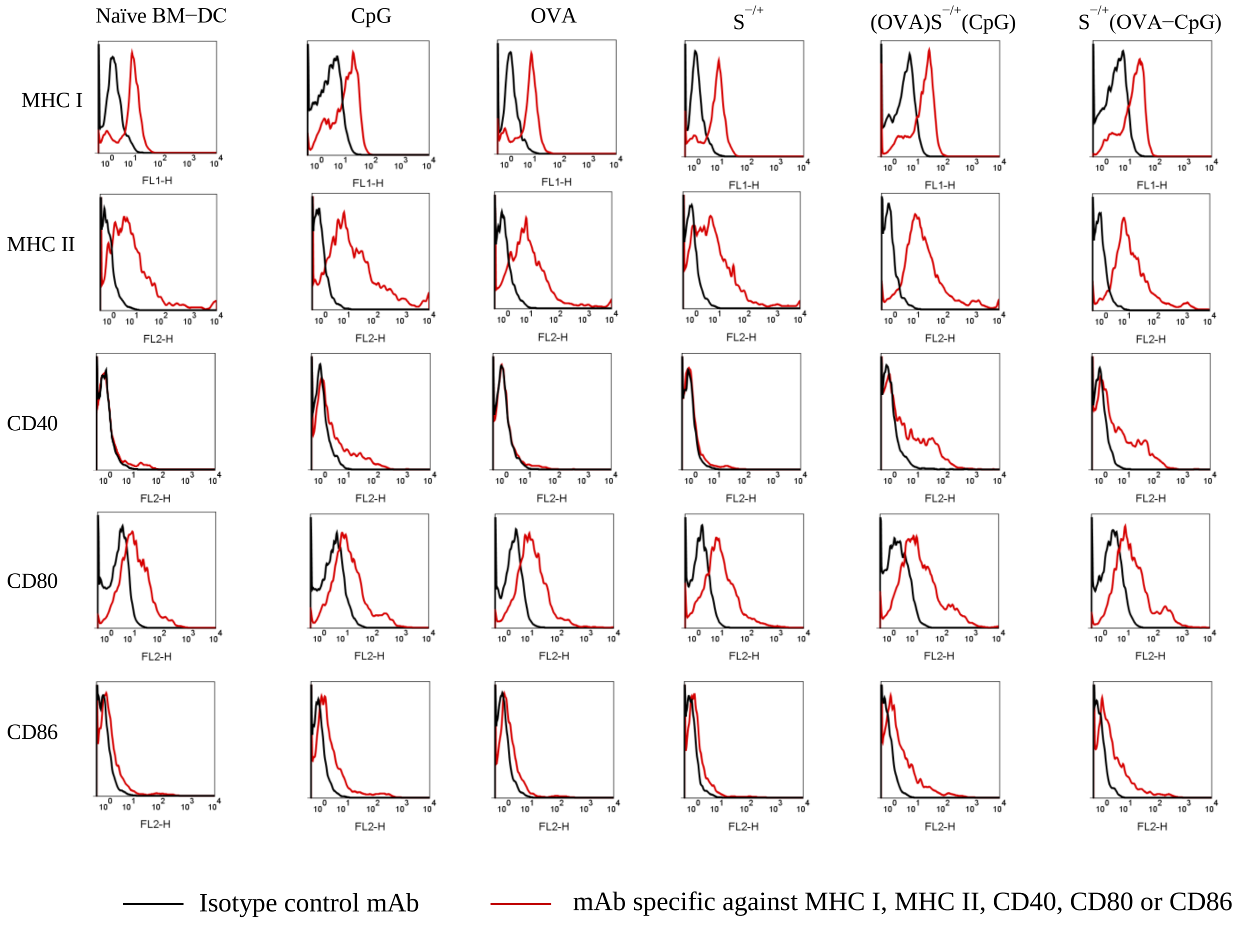

Figure S3. Effect of (OVA)S$^{-/+}$(CpG) or S$^{-/+}$(OVA−CpG) on BM−DC maturation *in vitro*. BM−DCs were incubated for 24 h with 5 µg/ml CpG, OVA, (OVA)S$^{-/+}$(CpG) or S$^{-/+}$(OVA−CpG), each contained 5 µg/ml OVA. BM−DCs were stained with fluorescently labelled specific antibodies against MHC I, MHC II, CD40, CD80 or CD86, and cell analysis was performed using flow cytometry. Representative histograms are shown for CD11c−expressing BM−DCs stained with specific fluorescently labelled antibodies or their corresponding isotype control antibodies.

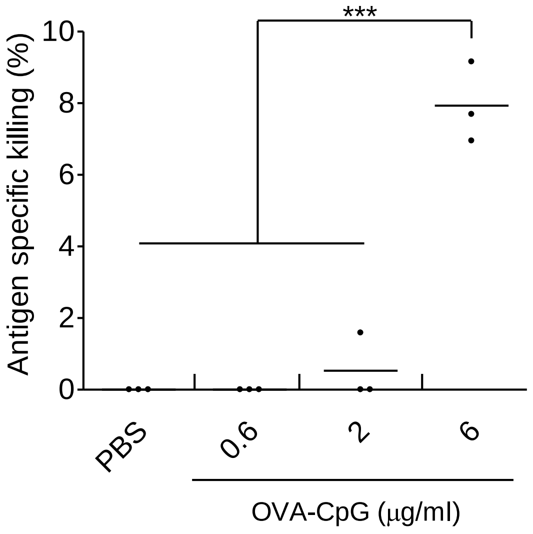

Figure S4. Determination of the optimal dose of OVA−CpG to induce antigen−specific killing using *in vivo* CTL assay. C57BL/6 mice (n= 3) were immunized, *via* footpad injection, with OVA−CpG containing 0.6, 2 or 6 µg of both OVA and CpG. On day 7 following immunization, a 1:1 splenocytes mixture consisting of target cells pulsed with 200 nM SIINFEKL and labelled with 0.5 µM CFSE (target cells) and unpulsed control cell labelled with 5 µM CFSE was intravenously administered to the control or immunized mice. Splenocytes were harvested, 18 hr later, from the control or immunized mice and analyzed using flow cytometry analysis. Antigen−specific killing induced by each treatment was determined. Each dot represents killing of target cells by each mouse, the mean value for each treatment is shown as a horizontal bar. *** P <0.001.

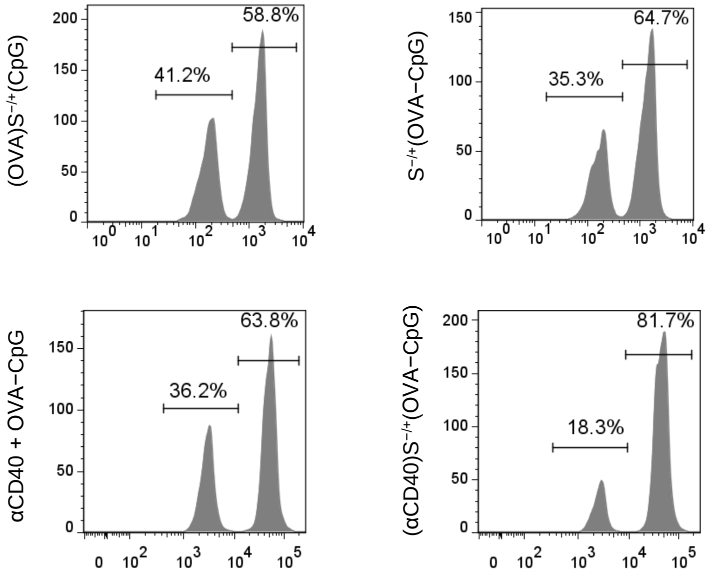

Figure S5. Assessment of *in vivo* CTL response. C57BL/6 mice (n= 3−5) were immunized, *via* footpad injection, with either 6 µg OVA (contained in (OVA)$S^{-/+}$(CpG) or $S^{-/+}$(OVA−CpG)) or 3 µg OVA (contained in OVA−CpG + αCD40 or (αCD40)$S^{-/+}$(OVA−CpG)). The $S^{-/+}$ unconjugated or conjugated αCD40 was used at 10 µg. On day 7 following immunization, a 1:1 splenocytes mixture consisting of target cells pulsed with 200 nM SIINFEKL and labelled with 0.5 µM CFSE (target cells) and unpulsed control cell labelled with 5 µM CFSE was intravenously administered to the control or immunized mice. Splenocytes were harvested, 18 hr later, from the control or immunized mice and analyzed using flow cytometry analysis. Representative histograms are shown for the detection of target and control cells, in the harvested splenocytes, using flow cytometry.

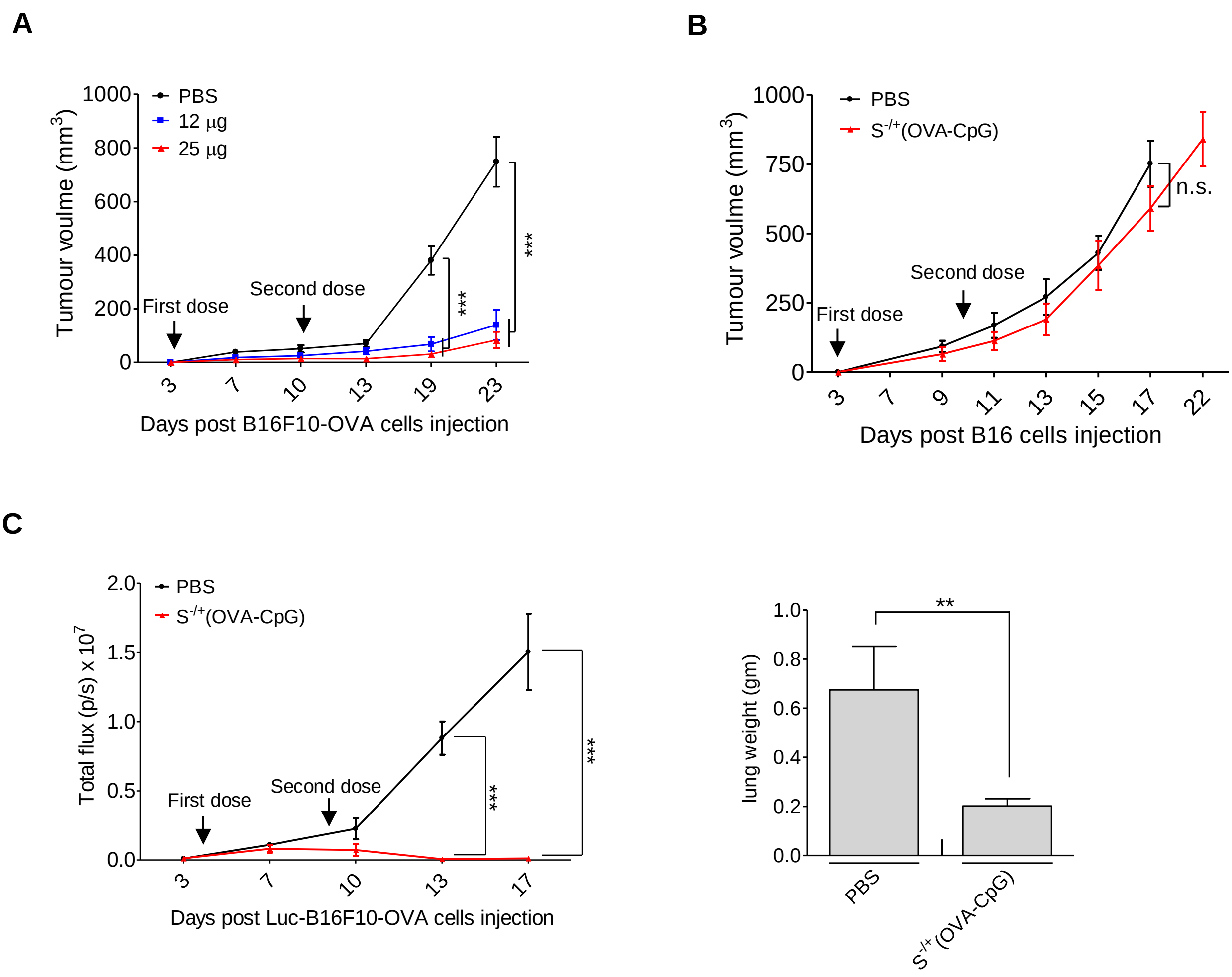

Figure S6. Assessment of anti−tumour immunotherapeutic efficiency of $S^{-/+}$(OVA−CpG) in subcutaneous or lung pseudo−metastatic tumour models. (A) C57BL/6 mice (n=8) were subcutaneously injected with 2.5×10^5 OVA−B16F10−Luc cells. On the 3rd and 10th days post tumour cells injection, tumour−inoculated mice were immunized *via* footpad injection with $S^{-/+}$(OVA−CpG) containing 12 or 25 µg OVA. Tumour volume was monitored using calliper measurement. Results are expressed as mean value ± SEM. **(B)** Anti−tumour immune response antigen specificity. C57BL/6 mice (n=8) were subcutaneously injected with 2.5×10^5 B16 cells. On the 3rd and 10th days post tumour cells injection, tumour−inoculated mice were immunized *via* footpad injection with $S^{-/+}$(OVA−CpG) containing 25 µg OVA. Tumour volume was monitored using calibre measurement. Results are expressed as mean value ± SEM. **(C)** Pseudo−metastatic lung tumour model. C57BL/6 mice (n=6−8) were intravenously injected with 2.5×10^5 OVA−B16F10−Luc cells. On the 4th and 9th days post tumour cells injection, tumour−inoculated mice were immunized *via* footpad injection with $S^{-/+}$(OVA−CpG) containing 12 µg OVA. Tumour growth was monitored using whole body imaging. (Left) Quantification of photon flux, expressed as number of photons per second (p/s). Values are expressed as mean value ± SEM. (Right) The weights of the lung excised from scarified tumour inoculated mice. Values are expressed as mean value ± S.D. ** $P < 0.01$, *** $P < 0.001$.

References

[1] Hassan HAFM, Smyth L, Rubio N, Ratnasothy K, Wang JTW, Bansal SS, et al. Carbon nanotubes' surface chemistry determines their potency as vaccine nanocarriers in vitro and in vivo. Journal of Controlled Release. 2016;225:205-16.
[2] Sloat BR, Sandoval MA, Hau AM, He Y, Cui Z. Strong antibody responses induced by protein antigens conjugated onto the surface of lecithin-based nanoparticles. Journal of controlled release : official journal of the Controlled Release Society. 2010;141:93-100.
